# Assessing development assistance for child survival between 2000 and 2014: A multi-sectoral perspective

**DOI:** 10.1371/journal.pone.0178887

**Published:** 2017-07-11

**Authors:** Chunling Lu, Annie Chu, Zhihui Li, Jian Shen, Subu Subramanian, Kenneth Hill

**Affiliations:** 1 Division of Global Health Equity, Brigham and Women’s Hospital, Boston, Massachusetts, United States of America; 2 Department of Global Health and Social Medicine, Harvard Medical School, Boston, Massachusetts, United States of America; 3 DST-NRF Center of Excellence in Human Development, University of Witwatersrand, Johannesburg, South Africa, Boston, Massachusetts, United States of America; 4 Department of Global Health and Population, Harvard School of Public Health, Boston, Massachusetts, United States of America; 5 Department of Family Medicine & Public Health, University of California, San Diego, California, United States of America; 6 Department of Social and Behavioral Sciences, Harvard School of Public Health, Boston, Massachusetts, United States of America; University of Hawaii at Manoa, UNITED STATES

## Abstract

**Background:**

The majority of Countdown countries did not reach the fourth Millennium Development Goal (MDG 4) on reducing child mortality, despite the fact that donor funding to the health sector has drastically increased. When tracking aid invested in child survival, previous studies have exclusively focused on aid targeting reproductive, maternal, newborn, and child health (RMNCH). We take a multi-sectoral approach and extend the estimation to the four sectors that determine child survival: health (RMNCH and non-RMNCH), education, water and sanitation, and food and humanitarian assistance (Food/HA).

**Methods and findings:**

Using donor reported data, obtained mainly from the OECD Creditor Reporting System and Development Assistance Committee, we tracked the level and trends of aid (in grants or loans) disbursed to each of the four sectors at the global, regional, and country levels. We performed detailed analyses on missing data and conducted imputation with various methods. To identify aid projects for RMNCH, we developed an identification strategy that combined keyword searches and manual coding. To quantify aid for RMNCH in projects with multiple purposes, we adopted an integrated approach and produced the lower and upper bounds of estimates for RMNCH, so as to avoid making assumptions or using weak evidence for allocation. We checked the sensitivity of trends to the estimation methods and compared our estimates to that produced by other studies. Our study yielded time-series and recipient-specific annual estimates of aid disbursed to each sector, as well as their lower- and upper-bounds in 134 countries between 2000 and 2014, with a specific focus on Countdown countries. We found that the upper-bound estimates of total aid disbursed to the four sectors in 134 countries rose from US$ 22.62 billion in 2000 to US$ 59.29 billion in 2014, with the increase occurring in all income groups and regions with sub-Saharan Africa receiving the largest sum. Aid to RMNCH has experienced the fastest growth (12.4%), followed by aid to Food/HA (9.4%), education (5.1%), and water and sanitation (5.0%). With the exception of RMNCH, the average per capita aid disbursed to each sector in the 74 Countdown countries was smaller than in non-Countdown countries. While countries with a large number of child deaths tend to receive the largest amount of disbursements, non-Countdown countries with small populations usually received the highest level of per capita aid for child survival among all 134 countries. Compared to other Countdown countries, those that met MDG 4 with a high reliance on health aid received much higher per capita aid across all sectors. These findings are robust to estimation methods.

**Conclusions:**

The study suggests that to improve child survival, better targeted investments should be made in the four sectors, and aid to non-health sectors could be a possible contributor to child mortality reduction. We recommend that future studies on tracking aid for child survival go beyond the health sector and include other sectors that directly affect child survival. Investigation should also be made about the link between aid to each of the four sectors and child mortality reduction.

## Introduction

As one of the Millennium Development Goals (MDG 4), reducing child mortality has been of top priority in the global health agenda. According to the World Health Organization (WHO), there were 75 countries known as the “Countdown countries”, that accounted for more than 90% of maternal and child deaths worldwide [1]. Only 25 of the Countdown countries reduced child mortality by two thirds from 1990 to 2015 [1]. Lack of sufficient financial support was one of the major barriers to scaling up cost-effective interventions for child survival in resource-poor settings [1–6]. Moving beyond 2015, child survival continues to lie at the center of the global health agenda: the Sustainable Development Goals (SDGs) advocate for all children to “have access to quality early child development, care and pre-primary education.” [7].

To inform stakeholders on how much more investments are needed for child survival, it is important to know, at both the global and country level, how much has been invested. Improving methods on tracking development assistance for child survival is important for promoting transparency and accountability, making evidence-based financial projections, and assessing the effectiveness of aid in improving child survival.

Exercises on tracking development assistance for child survival have been focused on health services for reproductive, maternal, newborn, and child health (RMNCH) [8–17]. Our study extends previous studies by taking a multi-sectoral perspective and expands estimation to food and humanitarian assistance, water and sanitation, and education. We adopted an integral approach in quantifying development assistance for RMNCH and examined aid for specific interventions. We imputed missing data and generated time-series and recipient-specific estimates on development assistance for child survival, as well as their lower- and upper-bounds, in 134 low- and middle- income countries between 2000 and 2014, with special attention to the 74 Countdown countries.

## Methods

### Conceptual framework for defining development assistance for child survival

**Fig 1:**
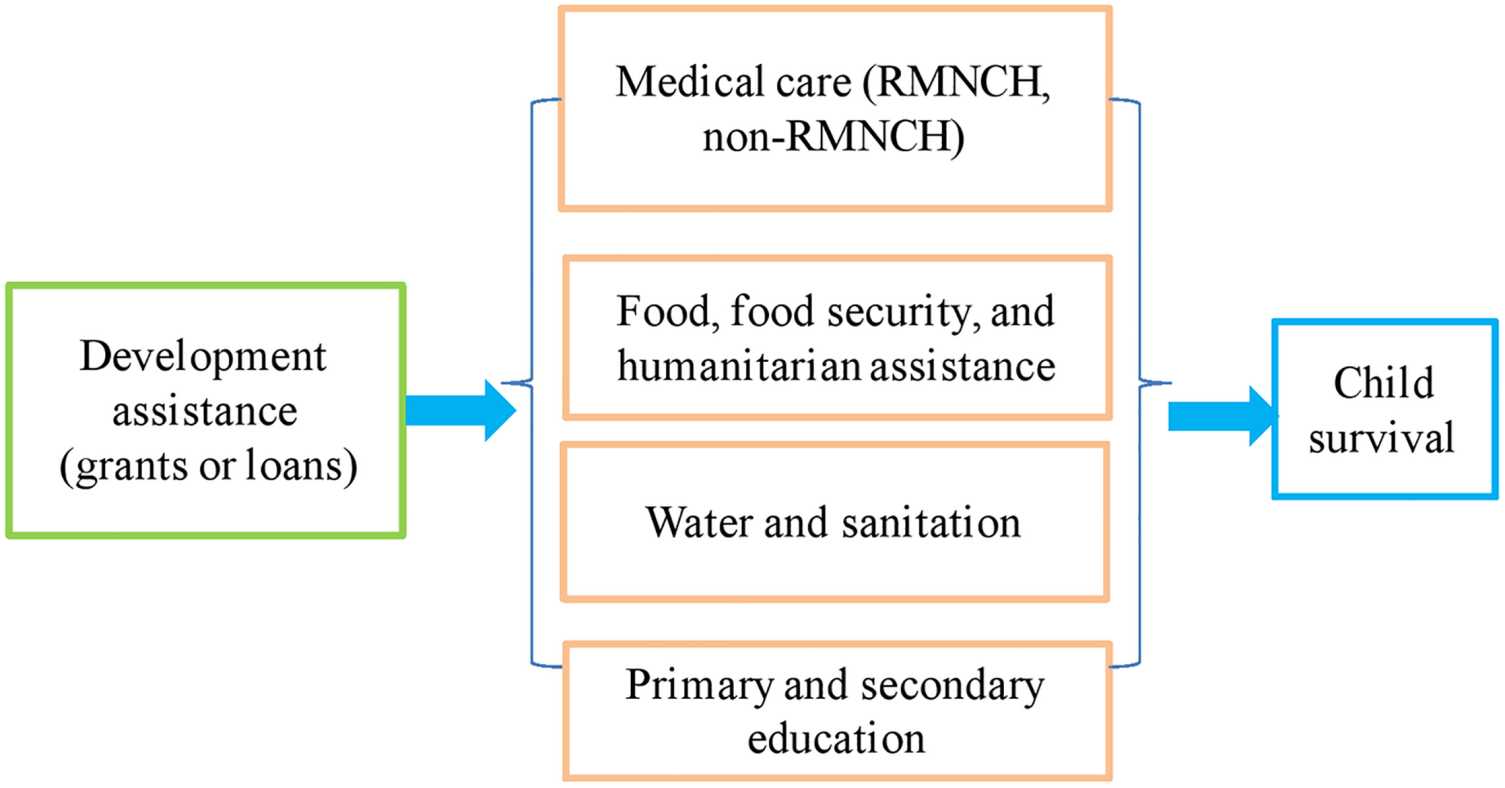
Conceptual framework on defining development assistance for child survival.

The definition of development assistance for child survival was built upon an influential and widely cited conceptual framework, proposed by Mosley and Chen [18], for the study of child survival in developing countries. The basic idea of the Mosley-Chen framework was that all background (social, economic, cultural, and health system) variables impacted child survival through a set of proximate determinants. Though the proximate determinants in different studies varied, the main categories included maternal health and education, environmental contamination (e.g., food security, water and sanitation), nutrient deficiency (calories, protein, micronutrients), and medical services (especially the RMNCH) [18–23]. Existing evidence from developing countries provided solid ground for the model and demonstrated that addressing the complexity of child mortality required joint and integrated efforts to improve these categories. Studies in developing countries showed that expanding access to primary and secondary schools greatly improved parental or maternal education and therefore improved child health outcomes [24]. In Zimbabwe, for example, an additional year of maternal secondary education was associated with 21% reduction in child mortality [25]. Unsafe drinking water and lack of sanitation accounted for 88% of global death from diarrhea [26]—a leading cause of death for under-five children [27]. Increasing access to clean water and sanitation effectively reduced child mortality and led to, for example, a 26% drop in child mortality in the poorest areas in Argentina [28]. WHO identified that about 45% of all child deaths were linked to malnutrition [27] and better nutrition for both mothers and children significantly reduced child mortality [29]. Based on the conceptual model and supporting evidence, our study defined development assistance for child survival as aid disbursed to proximate determinants: medical care (especially RMNCH); food, food security, and humanitarian assistance; water and sanitation; and primary and secondary education (**Fig 1**). According to this framework, aid to other sectors, such as agriculture or industry, operated through the proximate determinants to affect child survival, and therefore, was not included in our estimation. Measuring proximate determinants with the four areas was not exclusive, which is a limitation of this strategy. It is worth noting that the impact of aid to education on child survival through improving parental or maternal education may have a significant time lag.

There is no commonly-accepted approach on how to measure development assistance for RMNCH. Previous studies divided RMNCH into various categories and used different methods to classify the services in these categories. For example, the Institute for Health Metrics and Evaluation (IHME) provided mutually exclusive estimates on child care and maternal care, with little justifications offered [16]. Estimates on child and maternal care, produced by The Countdown Initiative, excluded projects on reproductive health and included a portion of expenditure on infectious disease control and health system strengthening [8–13], but their allocation of funds to child and maternal care relied on either assumptions or scanty evidence, which raised concern over their estimation [12, 17].

**Fig 2:**
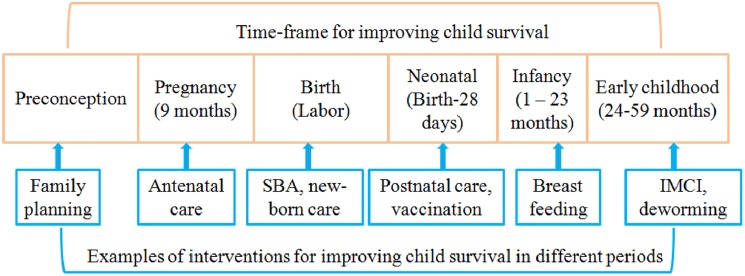
Conceptual framework on defining RMNCH.

In contrast to the differential approach adopted by previous studies, our study proposed an integral approach and defined development assistance for RMNCH as the aid for medical activities that have the purpose of preventing diseases, and restoring and improving RMNCH. Instead of dividing aid to RMNCH into different categories, we grouped all projects with activities on improving RMNCH into one category. Our approach was built upon the evidence that RMNCH are interdependent, and that interventions for maternal or reproductive health played a critical role for reducing still-births, and neonatal and infant death [30, 31]. The advantage of this approach is that we could avoid making assumptions on how funds were allocated across different categories for a multi-purpose project. The underlying message of the integral approach is that funds for interventions targeting different population groups are not competitive, but complementary or mutually supportive. Health interventions for RMNCH cover from pre-conception to pregnancy, to labor and delivery, to neonatal (birth to first month), to infancy (1–23 months), and early childhood (24–59 months), and include activities on RMNCH (**Fig 2**).

#### Data sources

To track the development assistance for child survival between 2000 and 2014, we used the aid datasets from the following sources: (1) The OECD Creditor Reporting System (CRS) aid activity database [32]. Data were downloaded in February 2016 with projects reported by 68 donors and implemented in 147 low and middle-income economies. We excluded 13 states or territories without complete time series data on total/child populations, or on child mortality rates, including South Sudan (a Countdown country). The final sample has 134 low- and middle-income countries (**S1 Text**). The CRS aid activity database documents aid activities reported by bilateral Official Development Assistance (ODA) to developing countries from OECD’s 26 member countries and European institutions, on a mandatory basis. It also provides information reported by multilateral organizations (such as the United Nations and World Bank), non-DAC countries (such as the Russian Federation, United Arab Emirates), and private donors (such as The Bill & Melinda Gates Foundation) on a voluntary basis (**S3 Table).**

(2) The OECD Development Assistance Committee (DAC) Annual Aggregate Database (DAC data). In addition to reporting to CRS aid activity database, DAC country members are required to report to DAC Annual Aggregates Database on their annual disbursements and commitments to recipients. The DAC2a database provides disbursements by a specific donor to a specific recipient in a specific year, yet has no breakdowns across sectors [33]. The DAC5 reported the level of disbursements (or commitments if disbursements were not available) for each sector by a donor in a specific year [34], but not by recipient countries. The reporting to CRS and DAC is conducted independently.

(3) Gavi, the Vaccine Alliance [35] and The Global Fund to Fight AIDS, Tuberculosis and Malaria (GFATM) [36]. The data from the GFATM and Gavi are not complete in CRS. We downloaded disbursements data between 2003 and 2006 from Gavi and 2002 from GFATM and added them to the CRS data.

### Estimating development assistance for child survival

We used disbursements (grants and loans) data to measure the development assistance for child survival between 2000 and 2014. Return of unspent balances and repayments of loans were excluded. Our focus is on the level of aid disbursed to developing countries rather than on comparing donors’ contribution patterns; therefore, donors without complete time series were included.

Based on the conceptual framework, we tracked the aid disbursed to projects with activities on improving RMNCH, water and sanitation, food and humanitarian assistance, and primary and secondary education in developing countries. **Table 1** presents the corresponding sector names and codes in the CRS data [37]. Aid that focused on assisting education/training, government and civil society, or other commodities could also include activities related to child survival. We used a list of key words to identify these activities and included them in the corresponding areas (**S3 Text**). Aid flows are measured on a calendar year basis.

**Table 1:** Sectors on determining child survival in the CRS database.

Sectors	Sector Code	Sector Name
Education	112, 113	Basic education, secondary education
Health	121, 122, 130	General health, basic health, reproductive health
Water and Sanitation	140	Water supply and sanitation
Food, food security, humanitarian assistance	520, 720, 730, 740	Food aid and food security, emergency response, reconstruction relief, disaster prevention and preparedness
**Other related sectors**	111, 114	Education and training in other levels
	150	Government and civil society
	530	Other commodity assistance

The CRS database has variables regarding project purpose, its title, donor(s), recipient(s), annual disbursements, and short and long descriptions of the project, which enabled us to derive the amount of aid disbursed by a donor in a year to a recipient country for activities directly related to RMNCH. We followed previous studies [14, 38] and used a combination of keyword search and manual coding, with keyword search as the first step and manual coding as the second step. Details on developing and implementing the identification strategies are presented in the **S3 Text**.

For projects with multiple purposes, two sets of estimates were generated: one including the full disbursements of multi-purpose projects (the upper-bound of estimated aid for RMNCH), and the other excluding multi-purpose projects (the lower bound of RMNCH).

### Imputing missing disbursements in CRS

One challenge of using the CRS data is the incompleteness of the reported disbursements, especially before 2003: donors reported aggregated disbursements to DAC, but did not report the related aid activities to the CRS. We analyzed and imputed the missing data and validated the imputation methods (**S4 Text**). The trends of missing rates suggested similar patterns across the four sectors: the missing rates are below 10% since 2008, except for food and humanitarian assistance between 2012 and 2014 (**S7 Fig)**.

Some donors reported disbursements only at the regional level or labeled it as “Developing countries, unspecified”. These unspecified funding could take a substantial proportion of total disbursements (33% in 2014, **S8 Fig**). We followed previous studies [12, 38] and allocated the annual regional or unspecified fund to each recipient based on its proportion in total aid disbursed to the region or to the developing countries in the year using available CRS data (**S4 Text**). All disbursements are deflated into 2013 US dollars.

#### Statistical analysis

We produced six sets of annual recipient-level estimates for aid disbursed to RMNCH (upper/lower bound), health, food and humanitarian, water and sanitation, and education (**Table 2**). For each sector, “CRS_rys_” represents the lowest value and “EST_rys_ + Est(Allo_rys__reg_unsp)” represents the highest value (**Table 2**). We tracked the levels and trends of aid for each child survival sector (in total and per capita) at the global and regional levels between 2000 and 2014, tested the robustness of trends, and examined their growth rates during the period.

**Table 2:** Six sets of annual recipient-level estimates.

Disbursements to a specific sector (s) in a specific year (y) to a specific recipient (r)	Data used
CRS_rys_	Non-missing CRS data
CRS_rys_+ Allo_rys__reg	Non-missing CRS data + regional funds allocated to a recipient
CRS_rys_+ Allo_rys__reg_unsp	Non-missing CRS data + regional and unspecified funds allocated to a recipient
EST_rys_	CRS_rys_+ imputed missing data
EST_rys_+ Est(Allo_rys__reg)	EST_rys_+ regional funds with imputed missing data allocated to a recipient
EST_rys_+ Est(Allo_rys__reg_unsp)	EST_rys_+ regional and unspecified funds with imputed missing data allocated to a recipient

To investigate whether resources were differentially allocated to the countries in high need (high child mortality rate), we estimated the aid per capita to each sector for countries with various characteristics: (1) 134 low- and middle-income countries; (2) 74 Countdown priority countries that accounted for more than 90% of child and maternal deaths worldwide [1]; (3) 67 Countdown countries with higher child mortality rate (greater than 40 per 1,000 live births) in 2000; (4) 25 fragile Countdown countries [39]; and (5) 15 Countdown countries that were on track to meet the MDG 4 and had a high-level of reliance on health aid (**S7 Table**). At the country level, we examined the total and per capita aid received during the period and compared the top 10 countries that received the largest amount of aid for each sector of child survival to the top 10 countries that received the highest per capita aid for each sector.

We also estimated trends for four types of interventions that targeted the leading causes of death for children under-five: (1) child vaccines and immunizations, (2) prevention and treatment of diarrhea and pneumonia, (3) prevention and treatment of malaria, and (4) services for neonatal health such as breastfeeding, antenatal care, neonatal care, postnatal care, prevention of mother-to-child transmission of HIV, and skilled birth attendance. Knowing how much aid is invested in these interventions could help both donors and recipients to identify underfunded services.

We compared our estimates of RMNCH in the Countdown countries with the ones produced by the Countdown group between 2003 and 2012 –the period with available estimates in the Countdown studies. STATA 14 was used in analysis.

## Results

### Aid disbursed to child survival sectors in 134 countries between 2000 and 2014

**Fig 3:**
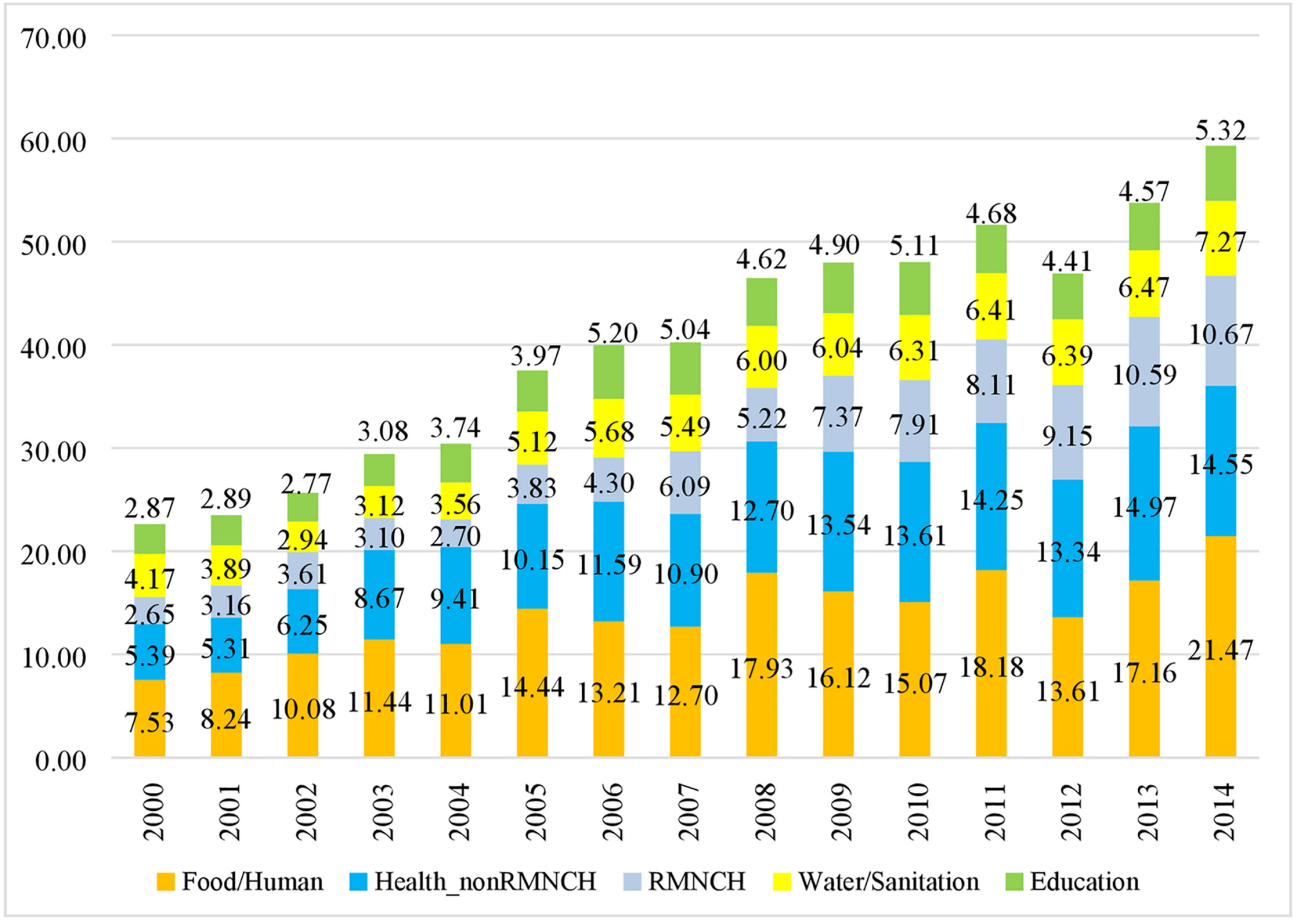
Upper-bound estimates of aid disbursed to RMNCH, health_non-RMNCH, food, water.

Despite a substantial difference in the level of the estimates before 2003, the general trends of global- and regional-level disbursements to each child survival sector do not seem to be sensitive to various estimation methods after 2003 (**S9 Fig and S10 Fig**). We present the highest value of the estimates “Est(Allo_rys__reg_unsp)” in the text. Between 2000 and 2014, the total amount of aid disbursed to the four sectors in the 134 countries more than doubled, rising from US$ 22.62 billion in 2000 to US$ 59.29 billion in 2014 (**Fig 3**) with an average growth rate of 7.4%. Per capita aid disbursed to the four sectors increased from US$ 4.64 to US$ 10.03 during the period. The health sector received the largest amount of disbursements each year, followed by food and humanitarian assistance, water and sanitation, and primary and secondary education (**Fig 3**). Increase in aid for child survival occurred in all income groups and regions, with Sub-Saharan Africa and low-income region receiving the largest amount of disbursements (**S11 Fig and S12 Fig).** Total aid to Countdown countries in each sector is larger than that in the non-Countdown countries (**S13 Fig**).

Within the health sector, aid for RMNCH quadrupled, from US$ 2.65 billion in 2000 (33% of total heath aid, $0.54 per capita) to US$ 10.67 billion in 2014 (42% of total health aid, $1.81 per capita) with an average annual growth rate of 12% (**Fig 3, Table 3, S14 Fig**). Over the 15 years, 48% of aid for RMNCH went to 10 Countdown countries: India, Nigeria, Ethiopia, Bangladesh, Pakistan, Tanzania, Kenya, Democratic Republic of Congo, Afghanistan, and Uganda (**S8 Table**). The average annual growth rate of aid is 12.4% and 8.1% for Countdown and non-Countdown countries, respectively.

**Table 3:** Upper-bound annual per capita disbursements to RMNCH, food and humanitarian assistance, education, and water/sanitation by different country groups, 2000–2014.

	2000	2001	2002	2003	2004	2005	2006	2007	2008	2009	2010	2011	2012	2013	2014	Avg.
***Per capita aid for RMNCH***																
134 countries	0.54	0.64	0.72	0.61	0.52	0.73	0.81	1.13	0.96	1.33	1.41	1.43	1.59	1.81	1.81	1.07
74 Countdown	0.56	0.66	0.72	0.62	0.53	0.77	0.86	1.19	1.02	1.43	1.52	1.54	1.7	1.95	1.95	1.13
60 non-Countdown countries	0.41	0.49	0.69	0.57	0.45	0.42	0.47	0.72	0.47	0.64	0.64	0.61	0.75	0.77	0.75	0.59
67 Countdown with high mortality states	0.87	1.03	1.12	0.95	0.83	1.2	1.34	1.85	1.6	2.2	2.33	2.38	2.63	3.02	3.01	1.76
25 Countdown fragile states	1.04	1.31	1.37	1.33	1.02	1.69	2.32	3.38	2.56	3.97	3.84	3.95	4.89	5.53	5.57	2.92
15 Countdown countries met MDG 4 & high reliance on health aid	2.27	2.38	2.61	2.55	1.98	3.18	4.18	5.46	4.63	5.71	6.11	6.64	7.17	8.61	8.69	4.81
***Per capita aid for health-non-RMNCH***																
134 countries	1.11	1.07	1.25	1.7	1.82	1.94	2.18	2.03	2.33	2.45	2.43	2.51	2.32	2.57	2.46	2.01
74 Countdown	1	1.01	1.12	1.45	1.69	1.81	1.98	1.91	2.22	2.37	2.44	2.52	2.36	2.57	2.33	1.92
60 non-Countdown countries	1.84	1.54	2.14	3.49	2.81	2.9	3.68	2.85	3.11	3.07	2.31	2.42	2	2.52	3.45	2.68
67 Countdown with high mortality states	1.5	1.53	1.63	2.07	2.52	2.72	2.89	2.81	3.32	3.47	3.63	3.74	3.5	3.86	3.5	2.85
25 Countdown fragile states	1.57	1.83	2.22	2.72	3.7	4.37	4.22	4.31	4.85	4.87	5.64	5.4	5.26	5.92	5.36	4.15
15 Countdown countries met MDG 4 & high reliance on health aid	5.19	4.94	4.63	6.82	8.25	7.7	10.16	8.97	10.53	11.13	12.16	12.17	11.36	12.05	11.52	9.17
***Per capita aid for food and humanitarian assistance***																
134 countries	1.55	1.67	2.01	2.25	2.13	2.76	2.49	2.36	3.29	2.92	2.69	3.2	2.36	2.94	3.63	2.55
74 Countdown	1.14	1.31	1.84	2.12	2.06	2.61	2.41	2.26	3.21	2.94	2.72	3.02	2.14	2.3	2.78	2.32
60 non-Countdown countries	4.44	4.18	3.24	3.18	2.64	3.85	3.07	3.11	3.82	2.73	2.44	4.54	4.06	7.77	10.04	4.21
67 Countdown with high mortality states	1.7	1.96	2.93	3.38	3.34	4.21	3.88	3.59	4.95	4.65	4.29	4.74	3.32	3.43	4.14	3.63
25 Countdown fragile states	2.85	3.89	6.93	8.6	9.14	10.62	10.6	9.99	14.45	13.31	12.71	13.53	8.7	8.5	9.54	9.56
15 Countdown countries met MDG 4 & high reliance on health aid	6.41	5.66	6.26	9.5	7.44	8.89	7.23	7.28	9.45	7.63	7.35	7.76	6.61	7.19	10.65	7.69
***Per capita aid for water and sanitation***																
134 countries	0.86	0.79	0.59	0.61	0.69	0.98	1.07	1.02	1.1	1.09	1.13	1.13	1.11	1.11	1.23	0.97
74 Countdown	0.71	0.67	0.52	0.49	0.59	0.9	0.96	0.91	0.96	0.93	0.98	1.01	0.99	1	1.09	0.85
60 non-Countdown countries	1.93	1.64	1.1	1.5	1.41	1.55	1.89	1.85	2.11	2.28	2.19	2.03	2.04	1.9	2.26	1.85
67 Countdown with high mortality states	0.88	0.78	0.67	0.64	0.8	1.23	1.37	1.23	1.16	1.18	1.28	1.28	1.34	1.27	1.35	1.1
25 Countdown fragile states	0.87	0.9	0.62	0.67	0.8	2.3	1.95	1.76	1.32	1.54	1.6	1.44	1.57	1.6	1.68	1.37
15 Countdown countries met MDG 4 & high reliance on health aid	2.67	2.29	1.84	2.19	2.24	2.4	3.77	2.93	2.64	2.87	2.81	2.88	3.37	3.49	3.17	2.77
***Per capita education aid***																
134 countries	0.59	0.58	0.55	0.61	0.72	0.76	0.98	0.94	0.85	0.89	0.91	0.82	0.77	0.78	0.9	0.78
74 Countdown	0.55	0.55	0.51	0.55	0.68	0.72	0.91	0.87	0.79	0.84	0.87	0.76	0.69	0.71	0.85	0.72
60 non-Countdown countries	0.84	0.82	0.87	0.98	1.02	1.06	1.47	1.45	1.27	1.22	1.26	1.28	1.34	1.36	1.3	1.17
67 Countdown with high mortality states	0.86	0.86	0.8	0.86	1.05	1.1	1.41	1.31	1.18	1.26	1.28	1.15	1.03	1.05	1.25	1.1
25 Countdown fragile states	0.93	0.86	0.69	0.85	0.98	1.19	1.17	1.83	1.26	1.42	1.67	1.31	1.5	1.51	1.73	1.26
15 Countdown countries met MDG 4 & high reliance on health aid	2.79	2.1	2.73	2.79	3.14	2.06	4.46	3.57	2.56	2.47	2.52	1.92	2.2	2.41	2.5	2.68
***Per capita aid for child survival (= health+education+water and sanitation+food and humanitarian assistance)***																
134 countries	4.64	4.75	5.12	5.78	5.89	7.17	7.53	7.48	8.52	8.68	8.57	9.09	8.15	9.21	10.03	7.37
74 Countdown	3.97	4.2	4.71	5.23	5.56	6.81	7.11	7.13	8.21	8.51	8.53	8.85	7.87	8.53	9	6.95
60 non-Countdown countries	9.46	8.67	8.04	9.73	8.32	9.76	10.58	9.97	10.79	9.95	8.84	10.88	10.19	14.32	17.8	10.49
67 Countdown with high mortality states	5.82	6.16	7.14	7.91	8.54	10.46	10.87	10.8	12.21	12.76	12.82	13.3	11.83	12.63	13.25	10.43
25 Countdown fragile states	7.26	8.79	11.84	14.17	15.64	20.19	20.26	21.27	24.44	25.12	25.47	25.63	21.91	23.06	23.88	19.26
15 Countdown countries met MDG 4 & high reliance on health aid	19.33	17.38	18.07	23.86	23.04	24.23	29.8	28.21	29.81	29.81	30.95	31.38	30.71	33.76	36.53	27.12

Food and humanitarian aid almost tripled, rising from US$ 7.53 billion ($1.55 per capita) in 2000 to US$ 21.47 billion ($3.63 per capita) in 2014 with an average growth rate of 9.4%. During the period, the top 10 countries (Sudan, Afghanistan, Ethiopia, Iraq, Pakistan, Syria, Democratic Republic of Congo, Somalia, Haiti, and Kenya) accounted for 48% of total aid flowing into this sector (**S8 Table**), and nine of them were Countdown countries. The average annual growth rate of aid is 9.9% and 12.5% for Countdown and non-Countdown countries, respectively.

Aid for water and sanitation grew from 4.17 billion ($0.86 per capita) in 2000 to 7.27 billion ($1.23 per capita) in 2014 with an average annual growth rate of 5.0%. During this period, the top 10 countries received largest amount of aid (India, China, Viet Nam, Iraq, Morocco, Tanzania, Bangladesh, Jordan, Indonesia, and Ethiopia) accounted for 35% of total aid in water and sanitation (**S8 Table**), and nine of them were Countdown countries. The average annual growth rate of aid is 5.8% and 3.6% for Countdown and non-Countdown countries, respectively.

Aid for education increased from US$ 2.87 billion ($0.59 per capita) in 2000 to 5.32 billion ($0.90 per capita) in 2014 with average growth rate of 5.1%. Over the 15 years, 42% of education aid went to 10 countries (India, Bangladesh, Pakistan, Afghanistan, Tanzania, Jordan, Indonesia, Ethiopia, Uganda, and Viet Nam (**S8 Table**), and nine of them were Countdown countries. The average annual growth rate of aid is 5.2% and 4.8% for Countdown and non-Countdown countries, respectively.

### Per capita aid disbursed to child survival sectors in 134 countries between 2000 and 2014

**Table 3** presents the trends of population-weighted annual per capita aid (upper-bound) for child survival by sector between 2000 and 2014. Except for RMNCH, the average annual per capita disbursement to each sector in the 74 Countdown countries is less than that in the 60 non-Countdown countries. Except for food and humanitarian assistance, the 15 Countdown countries with high reliance on health aid and on track to meet the MDG 4 received the highest level of per capital aid in each sector, followed by the 25 Countdown fragile countries and the 67 Countdown countries with high child mortality in 2000. The average per capita disbursements to the 15 Countdown countries are about four times of that for the 74 Countdown countries.

At the country level, the top 10 countries receiving the largest per capita aid in each sector included many with small populations not belonging to the Countdown group (e.g. Tuvalu, Micronesia, Tonga, Samoa, and Palau) (**S8 Table**). Annual per capita disbursements (upper-bound) to each sector of child survival are presented in **S9 Table**.

**Fig 4:**
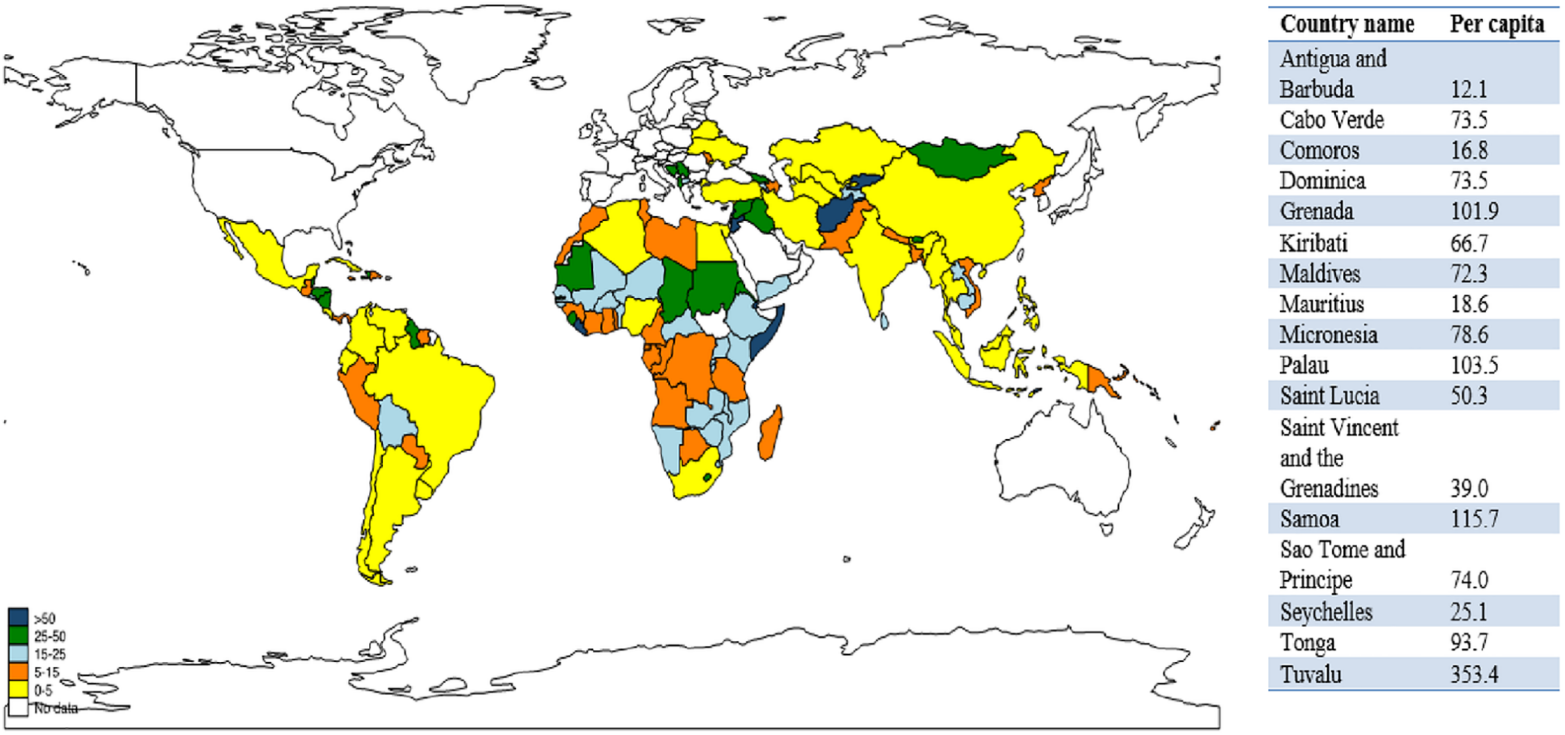
Upper-bound per capita aid for child survival in 134 countries over the 15-year period.

**Fig 4** summarizes the country-level per capita disbursements for child survival received by the 134 countries over the 15 years. We grouped the 134 countries into five groups according to the level of per capita aid received. The top group of 22 countries received US$50 or more per capita aid for child survival; most of them are either fragile states (e.g. Afghanistan, Somalia) or have small populations (e.g. Tuvalu, Samoa, Palau, Grenade, Micronesia); only seven of them belonged to the Countdown group. The second group of 22 countries received per capita aid between US$25 and US$50; only nine belonged to the Countdown group. The third group of 29 countries received per capita aid between $15 and $25. The fourth group of 33 countries received per capita aid between US$5 and US$15. The fifth group of 28 countries received less than US$5; and 12 are Countdown countries such as India, Indonesia, and Nigeria.

The East Asia & Pacific region had the highest mean per capita aid (US$51), followed by Middle East & North Africa (US$29), South Asia (US$25), sub-Saharan Africa (US$24), Latin America & Caribbean (US$20), and Europe & Central Asia (US$18). For individual countries in the same income group, per capita aid varied significantly. For instance, Tuvalu, a low-income country, received over US$353 per person per year over the period, whereas other low-income countries such as Bangladesh and Myanmar received less than US$10.

### Disbursements to interventions for RMNCH in the 74 Countdown countries

**Fig 5:**
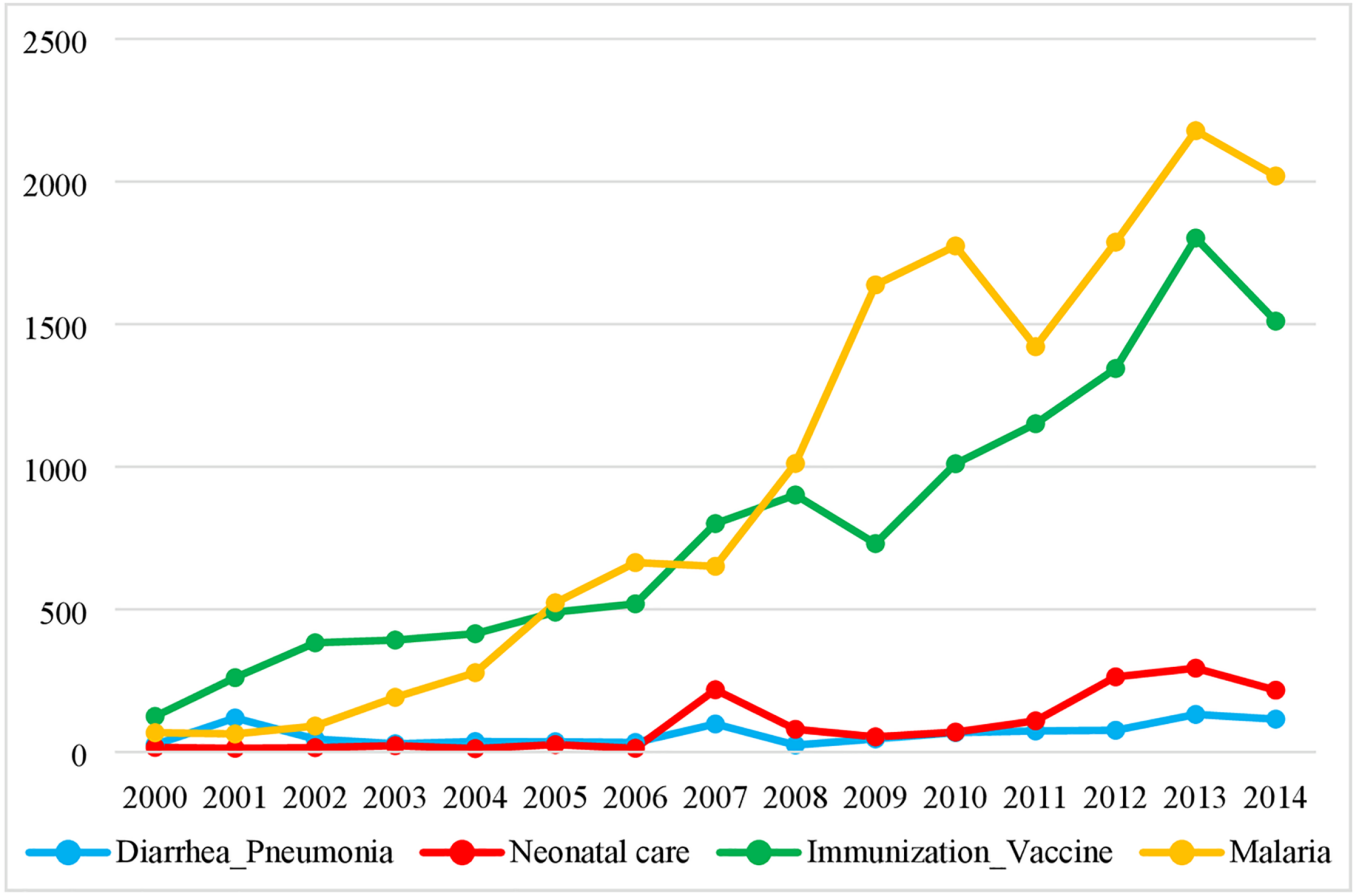
Upper-bound estimates of aid disbursed to projects with specific indications on listed.

Among the 74 Countdown countries, disbursements to malaria interventions and vaccinations increased sharply during the period, from US$ 67.42 million in 2000 to 2.02 billion in 2014 for malaria and from 124.5 million in 2000 to 1.51 billion in 2014 for vaccinations **(Fig 5**). Investments in preventing and treating diarrhea and pneumonia or promoting neonatal survival were relatively small (below US$30 million before 2007, and reaching its highest level in 2013 with 294 million). Aid to diarrhea and pneumonia increased from US$26 million in 2000 to US$115 million in 2014.

### Comparing estimates for RMNCH to the Countdown estimates for 74 Countdown countries, 2003–2012

**Table 4** presents the difference in definition, estimation methods, and data used between our study and the Countdown studies in tracking RMNCH. For example, our definition on RMNCH included projects with activities on reproductive health and nutrition, which were not included in the Countdown estimates. We did not allocate funds from general budget, for example to RMNCH. The integral approach enabled us to generate the upper- and lower-bound of aid disbursed to RMNCH. We used the CRS and DAC data downloaded in February 2016 and imputed missing data in CRS; and our study included 68 donors. The Countdown studies used CRS data downloaded at the different time point since 2005, and the 2015 study included 31 donors [12].

**Table 4:** Difference between our estimation and the countdown estimation.

	Our estimation	Countdown estimation
*Definition*	Projects with keywords directly related to child care, maternal and neonatal care, reproductive health, family planning, malaria, and nutrition	(1) Projects with keywords directly related to maternal, neonatal and child care + (2) proportion of HIV, TB, malaria, health system strengthening, general budget
*Estimation approach*	Integral	Differential
*Data used*	CRS, DAC, missing data imputed	CRS, missing data not imputed
*Time of data downloaded*	February, 2016	Different time point since 2005
*Donors included*	68 donors	31 donors
*Identification strategy*		Manual coding

**Fig 6:**
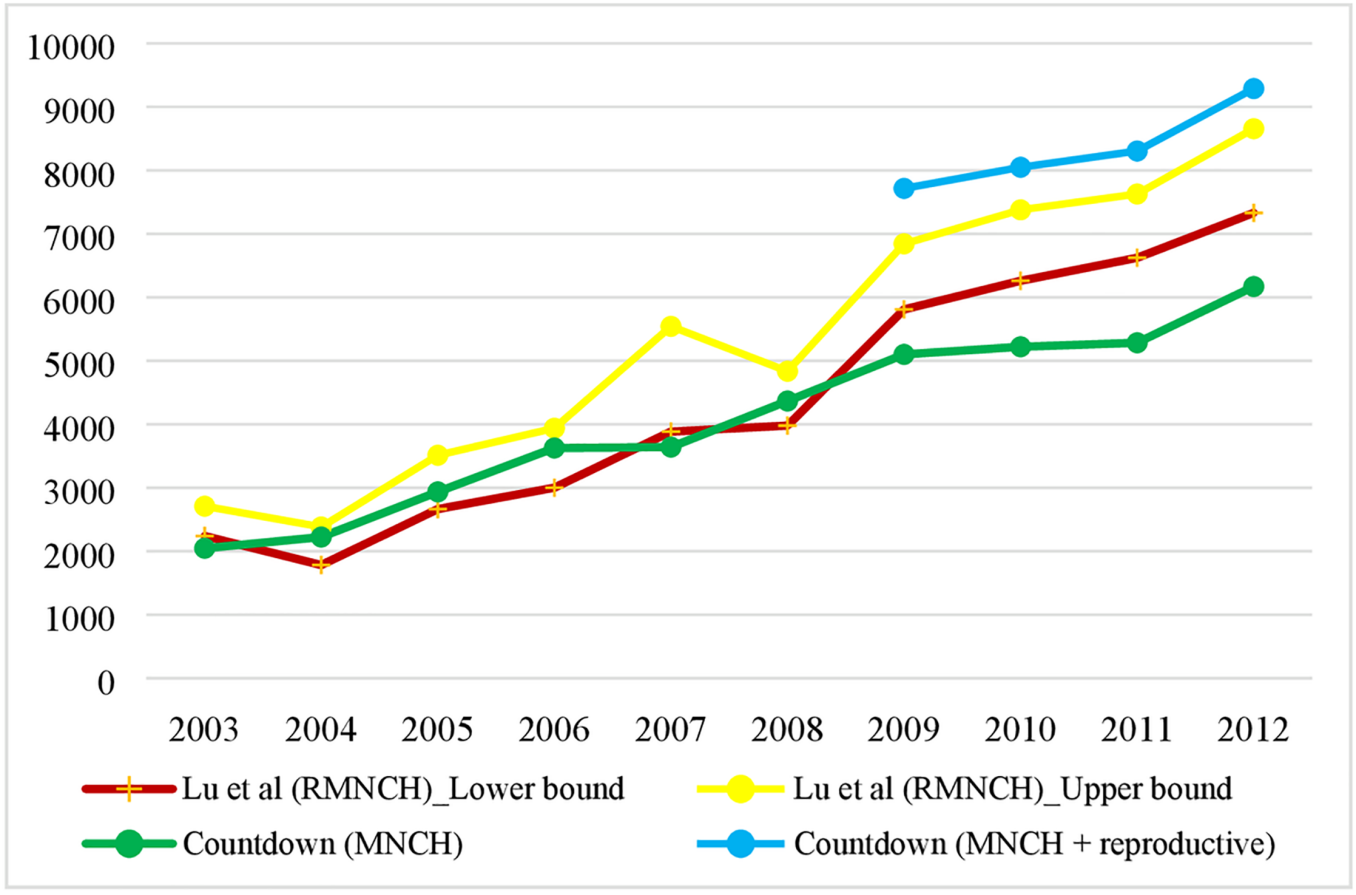
Comparison of estimates on RMNCH in the 74 Countdown countries in 2012 USD.

**Fig 6** shows the comparison of level and trends of the RMNCH estimates between our study and the Countdown studies. The qualitative results are consistent between the studies: aid for RMNCH has been increasing during the period, though the components in each estimate were not the same. Before 2009, the Countdown MNCH estimates were either close to or between our upper- and lower-bound of estimates of RMNCH. Since 2009, the Countdown MNCH estimates were below the lower bound of our estimates of RMNCH. After adding the Countdown’s estimates of reproductive health (available between 2009 and 2012) to its MNCH estimates, the Countdown’s RMNCH estimate were larger than our upper-bound estimate between 2009 and 2012. For example, in 2012, our upper-bound estimate for RMNCH was US$ 8.66 billion, and the Countdown’s RMNCH was US$ 9.29 billion.

## Discussion

Taking a multi-sectoral approach, we estimated aid disbursed to sectors that were considered as proximate determinants of child survival in the 134 countries between 2000 and 2014. We addressed missing data and methodological issues in assigning disbursements for multi-purpose projects to RMNCH. By analyzing the trends of the aid disbursed to each of the four sectors across different country groups, we have two salient findings. First, resource allocation clearly did not match well with the need during the period. Except RMNCH, the Countdown countries received smaller average per capita aid in each sector than the non-Countdown countries. Although countries with a large number of child deaths (e.g. India, China, Nigeria) or in conflict (e.g. Afghanistan, Iraq) tended to receive the largest amount of disbursements in each area, at the per capita level, countries with small populations usually received more aid per capita, and most of them did not belong to the Countdown group. Between 2000 and 2014, among the top 10 countries receiving the largest per capita aid for child survival, only one (Solomon Islands) belongs to the Countdown group.

Second, among the Countdown countries with high mortality rates, those meeting MDG 4 received much higher average per capita aid in each of the four sectors during the period: US$ 4.81 vs. US$ 1.76 for RMNCH, US$ 9.17 vs. US$ 2.85 for health-non-RMNCH, US$ 7.69 vs. US$ 3.63 for food and humanitarian assistance, US$ 2.77 vs. US$ 1.10 for water and sanitation, and US$ 2.68 vs. US$ 1.10 for primary and secondary education. Does the evidence imply that aid to health and non-health sectors in these 15 countries have worked together to bring down their child mortality rates? If the answer is yes, what was the contribution of each sector? For the countries that have not achieved MDG4, how much more should be invested in these sectors in order to meet the goal? To answer these questions, we need to go beyond the current study and assess the contribution of global specific aid to reducing child mortality using a multi-sectoral longitudinal analysis.

This study contributes to the body of knowledge in the following aspects. Firstly, we took a multi-sectoral perspective based on the child survival model that has been supported by evidence from both the developing countries, and extended the estimation of development assistance for child survival by including development assistance for food and humanitarian assistance, water and sanitation, and primary and secondary education. Secondly, to address methodological issues in quantifying developing assistance for RMNCH, we adopted an integrated approach and avoided making assumptions or using weak data to allocate funds for multi-purpose projects. We also developed an identification strategy, consisting of a combination of keyword searches and manually coding, to address the difficulty in replication of aid estimation for RMNCH. Thirdly, we analyzed the sources of missing data, conducted imputations, and validated imputation methods. We checked the sensitivity of the estimation. Fourthly, we conducted an in-depth study on development assistance for specific interventions including preventing and treating diarrhea, pneumonia, and malaria, expanding immunization, and providing antenatal, neonatal, and postnatal care.

However, this study has the following limitations. (1) Due to lack of data at the recipient level, our estimates did not capture aid from emerging economies to other low- and middle-income countries. Aid provided by NGOs and foundations (except the Bill & Melinda Gates Foundation) was also not included in the estimates. (2) While the combination of keyword search and manual coding increased the efficiency of identifying projects for child survival, the constructed keywords may not be able to capture all projects on child survival or those with typographical errors. In addition, the selection procedure relied purely on project descriptions, which could be poorly recorded by the donors. (3) There was a lack of data to provide information about domestic investments in child survival.

## Conclusion

This study estimated development assistance disbursed to the areas that were defined as proximate determinants of child survival in this study. The quality of estimates was improved with missing data addressed and upper and lower bounds obtained. While aid disbursed to child survival has been increasing in all income groups and regions between 2000 and 2014, better coordination among donors and recipients are required to improve the aid targeting. The estimates have also prepared us to conduct further investigations on the role of aid in reducing child mortality.

Going forward, on the research front, methods for effectively tracking aid to child survival should be discussed and agreed upon by different institutions. More critically, when defining aid for child survival, moving beyond the health sector and taking a multi-sectoral perspective are essential, particularly in the context of the SDGs era. In addition, aid effectiveness in reducing child mortality and its facilitators and barriers should be investigated and shared with policy makers and other stakeholders. On the policy front, improving child survival requires efforts from multiple sectors and needs strong political commitment. To scale up the proven cost-effective interventions in the Countdown countries, more domestic and foreign resources need to be raised. The launch of the Global Financing Facility [40], a new multi-stakeholder partnership for improving health of women, children, and adolescents, could set up a promising platform for accelerating global efforts in ending preventable child deaths through focusing on sufficient and sustainable financing.

## Supporting information

S1 Text Countries included in the study. (DOCX)

S2 Text Developing and implementing keyword search and manually coding. (DOCX)

S3 Text Analyzing and imputing missing data in the CRS. (DOCX)

S1 Table 134 low- and middle-income recipients in the CRS (according to the income group classification of the World Bank in 2013). (DOCX)

S2 Table Keywords for identifying projects for RMNCH, HIV/AIDS, TB, and health system strengthening. (DOCX)

S3 Table Donors reported to DAC2 and CRS and their available years. (DOCX)

S4 Table Websites and aid information for 8 donors. (DOCX)

S5 Table Root Mean Square Errors from the two methods. (DOCX)

S6 Table Countdown priority countries. (DOCX)

S7 Table Top 10 states receiving the largest amount of aid (upper-bound) over 15 years: total vs. per capita. (DOCX)

S8 Table Upper-bound per capita disbursements to each sector of child survival in 74 Countdown countries, 2000–2014. (DOCX)

S1 Fig Missing rate in disbursements in CRS among 134 low- and middle-income countries. (DOCX)

S2 Fig Missing rate for disbursements by income group in CRS. (DOCX)

S3 Fig Missing rate for disbursements by region in CRS. (DOCX)

S4 Fig Missing rates for Countdown countries with different reliance level on health aid. (DOCX)

S5 Fig Sources of missing components in low- and middle-income between 2000 and 2014 (in millions of 2013 USD). (DOCX)

S6 Fig Process of validation. (DOCX)

S7 Fig Estimated percentage of missing disbursements data in sectors. (DOCX)

S8 Fig % of disbursements with recipients as “regional” or “unspecified”. (DOCX)

S9 Fig Global trends of aid disbursed to health, RMNCH, food and humanitarian assistance, water and sanitation, and education in 134 countries with six sets of estimates, 2000–2014 (in millions of 2013 USD). (DOCX)

S10 Fig Regional trends of aid disbursed to health, RMNCH, food and humanitarian assistance, water and sanitation, and education in 134 countries with six sets of estimates, 2000–2014 (in millions of 2013 USD). (DOCX)

S11 Fig Upper-bound estimates of aid disbursed to sectors for child survival in millions in 2013 USD by region, 2000–2014. (DOCX)

S12 Fig Upper-bound estimates of aid disbursed to sectors for child survival in millions in 2013 USD by income, 2000–2014. (DOCX)

S13 Fig Upper-bound estimates of aid disbursed to RMNCH, health_non-RMNCH, food, water and sanitation, humanitarian assistance, and education in billions in 2013 USD, Countdown vs. non-Countdown, 2000–2014. (DOCX)

S14 Fig Proportion of aid (upper-bound) for RMNCH in total health aid. (DOCX)
