# Correction: Assessing development assistance for child survival between 2000 and 2014: A multi-sectoral perspective

**DOI:** 10.1371/journal.pone.0192309

**Published:** 2018-01-30

**Authors:** Chunling Lu, Annie Chu, Zhihui Li, Jian Shen, S. V. Subramanian, Kenneth Hill

The fifth author's name is incorrect. The correct name is: S. V. Subramanian. The correct citation is: Lu C, Chu A, Li Z, Shen J, Subramanian SV, Hill K (2017) Assessing development assistance for child survival between 2000 and 2014: A multi-sectoral perspective. PLoS ONE 12(7): e0178887. https://doi.org/10.1371/journal.pone.0178887
