# Supplementary material for: Assessing development assistance for child survival between 2000 and 2014: A multi-sectoral perspective
Source: PLoS One. 2017 Jul 11;12(7):e0178887. doi: 10.1371/journal.pone.0178887 (PMC5507412; doi:10.1371/journal.pone.0178887)
Supplement: S1 Text — (DOCX) [file pone.0178887.s001.docx]

**S1 Text. Countries included in the study**

We included 134 low- and middle-income countries, classified by the World Bank in 2013, in our study. There are 51 low-income countries, 33 lower-middle income countries, and 50 upper-middle income countries. 13 countries were excluded due to lack of complete time-series data on total population, or child population, or child mortality. Most of them are countries with small population size (**S1 Table**).
