# Supplementary material for: Assessing development assistance for child survival between 2000 and 2014: A multi-sectoral perspective
Source: PLoS One. 2017 Jul 11;12(7):e0178887. doi: 10.1371/journal.pone.0178887 (PMC5507412; doi:10.1371/journal.pone.0178887)
Supplement: S2 Text — (DOCX) [file pone.0178887.s002.docx]

**S2 Text. Developing and implementing keyword search and manually coding**

Previous studies identifying aid for child and maternal health relied on keywords search through these variables^1-3^ or manually coding based on a framework.^4-9^ Both have their strengths and weakness as analyzed in Pitt et al^1^: while manually coding each project could be time-consuming and hard to replicate, keywords search could lead to systematic misclassification of projects. In our previous study on development assistance for mental health,^3^ we followed Pitt et al.^1^ and used a combination of keyword search and manually coding to correct for misspecifications, with keyword search as the first step and manually going through all projects that survived the keywords search as the second step. In this study, we used the same approach to identify projects for health services for reproductive, maternal, newborn, and child health (RMNCH).

Since the keywords may not be able to capture all of the projects for RMNCH, the approach may not eliminate the possibility of excluding projects targeting RMNCH. To examine how many projects for RMNCH were not captured by the keyword search, we randomly selected 10% of the projects (2,084) from the health sector data in 2011 and applied both keywords search and manually coding to the selected projects. We compared the results from the two methods and found that the keyword search approach missed about 1·1% of projects for RMNCH (366), suggesting keyword search efficient and time saving.

We constructed the keywords to identify projects targeting under-five child and women before, during, and after pregnancy. We started with a group of words to identify the target populations, such as “newborn”, “neonatal” “infant”, “child”, “maternal”, “reproductive health”, “pregnant”, etc. Based on the lead causes of child and maternal deaths, we then produced keywords on conditions and diseases, such as “abortion”, “birth”, “diarrhea”, “pneumonia”, “prematurity”, “post-partum haemorrhage”, etc. Keywords on interventions or programs were constructed based on those used in previous studies as well as literature review,^1-3, 10-30^ including “skilled-birth attendance”, “antenatal/neonatal/postnatal care”, “breast-feeding”, “oral rehydration solution”, “zinc therapy”, “immunization”, etc. Malnutrition and malaria have been among the leading causes for under-five mortality and their treatment is a component of Integrated Management of Childhood Illness, we also included them in child and maternal care category.

A full list of keywords can be found in **S3 Table**. To capture projects reported with other languages, we translated the keywords into Dutch, French, Germen, Italian, and Spanish using Google Translator. We used the STATA 14 to conduct keyword search on four variables in the CRS: a project’ purpose, its title, and short and long descriptions. Projects with purposes on family planning, reproductive health or projects funded by GAVI, UNICEF, and UNFPA were considered for RMNCH. If a project has one of the keywords in its title, or short/long descriptions, we assigned this project as RMNCH. For a project with multiple keywords, (e.g., projects providing oral rehydration solution for patients with diarrhea), we chose one of keywords to avoid double counting. Without compromising specificity, we also truncated the key words to make it more general to capture the related projects.

Note that one project may have activities with multiple purposes. For example, the United States supported a project on purposes of reducing transmission of HIV/AIDS and improving reproductive, maternal and child Health. Previous studies split the aid evenly across these purposes so they can be mutually exclusive^2^ or allocated the funds based on weak or outdated data factors obtained from the literature review.^4-9^ In this study, we took a different approach and generated two sets of estimates for RMNCH: one including full disbursements of a project with multiple purposes if it contains a keyword for RMNCH (the upper bound), and the other excluding projects with activities on HIV/AIDS or TB or health system improvements even though they have keywords for child and maternal care (the lower bound).

We applied the keywords search to over 307,830 projects in health sector between 2000 and 2014 and obtained 116,081 projects for RMNCH in the upper bound. When calculating aid for RMNCH in the lower bound, about 23% of projects were excluded from the upper bound. Two analysts were assigned to go through these projects independently and manually reviewed each project and corrected the misclassified projects. For example, we found that the keyword “child” captured school children who were older than 5 years. We excluded them from the estimates. For those without clear indication of their age, we assumed that they were younger than 5 years.

**References**

Pitt C, Lawn JE, Ranganathan M, Mills A, Hanson K. Donor funding for newborn survival: an analysis of donor-reported data, 2002–2010. *PLoS Med* 2012; **9:** e1001332.

Sources and Focus of Health Development Assistance, 1990–2014. *JAMA*. 2015;313(23):2359-2368. doi:10.1001/jama.2015.5825

Gilbert BJ, Patel V, Farmer PE, Lu C. Assessing Development Assistance for Mental Health in Developing Countries: 2007–2013. PLoS Med 2015;12(6): e1001834. doi:10.1371/journal.pmed.1001834

Powell-Jackson T, Borghi J, Mueller DH, Patouillard E, Mills A. (2006) Countdown to 2015: tracking donor assistance to maternal, newborn and child health. Lancet. 368(9541):1077–1087. pmid:16997662 doi: 10.1016/s0140-6736(06)69338-0

Greco G, Powell-Jackson T, Borghi J, Mills A. (2008) Countdown to 2015: Assessment of Donor Assistance to Maternal, Newborn, and Child Health between 2003 and 2006. Lancet. 371(9620):1268–1275. doi: 10.1016/S0140-6736(08)60561-9. pmid:18406861

Pitt C, Greco G, Powell-Jackson T, Mills A. (2010) Countdown to 2015: assessment of official development assistance to maternal, newborn, and child health, 2003–08. Lancet. 376(9751):1485–1496. doi: 10.1016/S0140-6736(10)61302-5. pmid:20850869

Hsu J, Pitt C, Greco G, Berman P, Mills A. Countdown to 2015: changes in offcial development assistance to maternal, newborn, and child health in 2009–10, and assessment of progress since 2003. *Lancet* 2012; **380:** 1157–68

Hsu J, Berman P, Mills A. Reproductive health priorities: evidence from a resource tracking analysis of official development assistance in 2009 and 2010. *Lancet* 2013; **381:** 1772–82.

1. Arregoces L, Daly F, Pitt C, Hsu J, Martinez-Alvarez M, Greco G, Mills A, Berman P, Borghi J. Countdown to 2015: changes in official development assistance to reproductive, maternal, newborn, and child health, and assessment of progress between 2003 and 2012. *Lancet Glob Health* 2015;3: e410–22

## A Decade of Tracking Progress for Maternal, Newborn and Child Survival: The 2015 Report. http://www.countdown2015mnch.org/reports-and-articles/2015-final-report

1. http://www.who.int/topics/millennium_development_goals/child_mortality/en/
2. http://www.who.int/pmnch/en/
3. Bhutta ZA, Chopra M, Axelson H, et al. Countdown to 2015 decade report (2000–10): taking stock of maternal, newborn, and child survival. *Lancet* 2010; **375:** 2032–44.
4. Darmstadt GL, Bhutta ZA, Cousens S, Adam T, Walker N, et al. (2005) Evidence-based, cost-effective interventions: how many newborn babies can we save? Lancet 365: 977–988.
5. Bhutta ZA, Das JK, Walker N, Rizvi A, Campbell H, Rudan I, et al. Interventions to address deaths from childhood pneumonia and diarrhoea equitably: what works and at what cost? The Lancet. 2013;381(9875):1417-29.
6. Bhutta ZA, Das JK, Bahl R, Lawn JE, Salam RA, Paul VK, et al. Can available interventions end preventable deaths in mothers, newborn babies, and stillbirths, and at what cost? The Lancet. 2014;384(9940):347-70.
7. <https://blogs.unicef.org/blog/why-early-childhood-development-is-the-foundation-for-sustainable-development/>
8. Mosley WH, Chen LC. 1984. An analytic framework for the study of child survival in developing countries. Population and Development Review 10:25-45.
9. Hill K, Pebley AR. 1989. Child mortality in the developing world. Population and Development Review 15(4):1657-687.
10. Hill K. Frameworks for studying the determinants of child survival. 2003. Bulletin of World Health Organization 81 (2):138-139.
11. Wolpin, K., 1997. Determinants and consequences of the mortality and health of infants and children. In: Rosenzweig, M., Stark,O. (Eds.), Handbook of Population and Family Economics. Elsevier, Amsterdam.
12. Barrera, A. 1990. The role of maternal schooling and its interaction with public health programs in child health production. Journal of Development Economics 32:69–91
13. [Karen A. Grépin](http://www.sciencedirect.com/science/article/pii/S0167629615000867)^,^[Prashant Bharadwaj](http://www.sciencedirect.com/science/article/pii/S0167629615000867) , Maternal education and child mortality in Zimbabwe. JHE 2015 December
14. <http://www.cdc.gov/healthywater/global/wash_statistics.html>
15. <http://www.who.int/mediacentre/factsheets/fs178/en/>
16. Galiani, S., Gertler, P., Schargrodsky, E., 2005.Water for life: the impact of the privatization of water services on child mortality. Journal of Political Economy 113: 83–120.
17. Hanson MA, Bardsley A, De-Regil LM, Moore SE, Oken E, Poston L, et al. The International Federation of Gynecology and Obstetrics (FIGO) recommendations on adolescent, preconception, and maternal nutrition: “Think Nutrition First”#. International Journal of Gynecology &Obstetrics. 2015;131:S213.
18. Imdad A, Yakoob MY, Bhutta ZA. The effect of folic acid, protein energy and multiple micronutrient supplements in pregnancy on stillbirths. BMC Public Health. 2011;11(Suppl 3):S4.
19. Zimmermann MB. The effects of iodine deficiency in pregnancy and infancy. Paediatric and perinatal epidemiology. 2012;26(s1):108-17.
20. The Partnership for Reproductive, Maternal, Newborn & Child Health. Essential Interventions, Commodities and Guidelines for RMNCH. A Global Review of the Key Interventions Related to Reproductive, Maternal, Newborn and Child Health (RMNCH). Geneva, PMNCH, 2011
