# Supplementary material for: Assessing development assistance for child survival between 2000 and 2014: A multi-sectoral perspective
Source: PLoS One. 2017 Jul 11;12(7):e0178887. doi: 10.1371/journal.pone.0178887 (PMC5507412; doi:10.1371/journal.pone.0178887)
Supplement: S3 Text — (DOCX) [file pone.0178887.s003.docx]

**S3 Text. Analyzing and imputing missing data in the CRS**

1. Analyzing missing aid activity data in CRS

The CRS aid database can be used to obtain yearly disbursements to recipient countries by sectors. One major issue of the CRS data is its incompleteness of the reported disbursements. Some donors reported the aggregated disbursements to DAC, but did not report the related aid activities to the CRS. The difference between disbursements reported to DAC and CRS has been used to measure the completeness of CRS data: the missing rate in each year is the difference between aggregated disbursements reported to the CRS and to the DAC as a percentage of aggregated disbursements reported to the DAC. According to the DAC, overall missing rate in CRS is about 40% before 2002 and the completeness rate reached nearly 100% since 2007.^1^ Missing rates at the sector level, however, were not available. It is not known whether the missed disbursements data in CRS is randomly distributed across the sectors or focused in a few sectors.

We started with examining the missing activity data in 134 low- and middle- income recipients in the CRS between 2000 and 2014. We found that, the missing rate decreased from 53% in 2000 to 5% in 2006, and rose up in 2007 to 15%, and then went down to 9% or below since 2008 (**S1 Fig.**).

We then examined the missing rates by income groups and regions to check whether countries in a specific region or income group were more likely to have missing data than others. For three income groups, except lower-middle income group, the missing rate went down to less than 10% since 2008 (**S2 Fig.**).

Missing rates in all regions improved substantially and were 10% or below since 2006 except a few exceptions: in America, the missing rate jumped back to 44% in 2007 due to lack of reporting in the CRS from IDB Special Fund on disbursements to Nicaragua and Bolivia. In Asia, the missing rates were 14% and 12% in 2007 and 2009 respectively (**S3 Fig).** Data missing in African region has the biggest improvement: missing rate went down from 54% in 2000 to 8% or below since 2005.

Note that the same missing rate in the two countries with different level of reliance on foreign aid means differently for the two countries. With available country-level health expenditure data in health sector,^2^ we obtained annual average missing rate between 2000 and 2014 for 74 priority countries listed in the Countdown Initiative with different level of reliance on health aid, measured by % of health aid in total health expenditure. We chose 10% as a cutoff point for high- or low-reliance on health aid: among 134 countries, the median of government spending on health as % of government spending is 10%. The average missing rate for high-reliance countries is very close to that for low-reliance countries (**S4 Fig.**), suggesting no systematic difference in missing pattern between these two groups. Analysis about aid effect on child mortality should be conducted separately for the countdown priority countries with different reliance on health aid.

We further identified the sources of missing data by comparing disbursements reported by a specific donor (d) to a specific recipient (r) in a year (y) in the DAC (DAC_dry_) to that in CRS (CRS_dry_). Our investigation shows that the difference between the data in DAC and CRS mainly comes from three channels: (1) donors reported to DAC but not to CRS, (2) donors reported to both CRS and DAC with more years or more recipients in DAC than in CRS, and (3) donors reported to both CRS and DAC in the same year for the same recipients, but the level in CRS is smaller than that in the DAC.

There are 84 donors reported to the DAC and among them, 67 donors reported aid activities to the CRS. The 17 donors who reported to DAC but not to CRS include Saudi Arabia, Russia, Israel, United Nations Regular Programme for Technical Assistance (UNTA), Chinese Taipei, etc. Among 67 donors reported to both CRS and DAC, 25 of them have more years reported to the DAC database. For example, Czech Republic had annual disbursement data between 2000 and 2014 in DAC but it only reported to CRS between 2011 and 2014 in CRS. World Food Programme has full range of annual disbursements data between 2000 and 2014 in DAC but only reported to CRS between 2008 and 2014. Food and Agriculture Organization reported to CRS (in 2013) but not to DAC (**S4 Table**).

Between 2000 and 2014, the proportion of missing component from source 1 increased from 2% in 2000 to 66% in 2014; proportion of source 2 increased from 11% in 2000 to 32% in 2008 and then decreased to 3% in 2014; proportion of source 3 (the largest source of missing before 2012) decreased from 88% in 2000 to about 30% in 2014 (**S5 Fig.**). As the completeness of data reporting from traditional donors has improved over time, emerging donors (such as Russia, etc.) has become the main source for data missing.

1. Addressing missing aid activity data in CRS in previous studies

There are three main approaches in dealing with missing disbursements data in the CRS. To avoid high level of missing disbursements between 2000 and 2002, the Countdown group conducted estimation since 2003. In their 2008 study (covering 2003 to 2006 period), they “corrected for missing data over the 4 years by dropping those donors that did not report so as to ensure that trends were comparable and were not affected by changes in reporting status.”^3^ Our calculation showed that the missing rate in 2003 and 2004 were 18% and 17% (**S1. Fig.),** respectively. About 40% and 45% of projects reported by the United States (the largest bilateral donor) had missing disbursements in the health sector in these two years. The estimates produced by the Countdown in these years could be substantially underestimated by its definition.

The study by Ravishankar et al^4^ imputed a donor’s yearly missing disbursements by using its commitment data and a 6-year project-level average disbursement schedule - constructed from a donor’s existing project-level disbursements and commitments by assuming that every project lasted for 6 years and a donor applied the same disbursement schedule to all recipients. While actual projects may be shorter or longer than 6 years, taking no consideration of heterogeneity of the disbursement schedule across recipients could disturb aid estimates at the recipient level. We used the CRS data for the United States in 2011 (the year with the lowest missing rate), and calculated the ratio of disbursements to commitments by recipient countries in health sector. The ratio ranged from 0 to 4·33 and has median 0·83 and inter-quartile range between 62% and 1, suggesting a large variation in disbursement schedule at the recipient level.

The Van de Sijpe’s method^5^ adopted a new perspective and imputed missing data in the CRS using information from DAC2. The study calculated the difference in disbursements data between DAC2 and CRS at the donor, recipient, and year level (DIF_dry_) and imputed the DIF_dry_ at the sectoral level for adding back to the CRS_dry_ at the sectoral level. Van de Sijpe’s study used DAC5 which has breakdowns of commitments (or disbursements if commitments are not available) at the donor, year, and sectoral level, but not at recipient level. The study constructed the sector share from DAC5 for each donor and year and multiplied the share to DIF_dry_ for obtaining DIF_dry_ in each sector. The study assumed that a donor in a specific year disbursed the same share of its funds to a specific sector across all recipient countries. We used the CRS’ disbursements data from the United States in 2011 and calculated its share for disbursements in health sector across 134 recipients: the health sector share is between 0 and 1 with median 0·17 and inter-quartile range between 0·03 and 0·37, suggesting a large variation in sectoral share across recipients for a donor in a year.

1. Imputing missing disbursements data in the CRS

Using donor information to estimate the missing disbursements may not distort global- or regional-levels of estimates. As our focus is on country-level estimates of disbursements for child survival, it is necessary to take into consideration recipients’ information in the estimation. We followed the perspective of the Van de Sijpe’s study, but using both donor and recipient’s information (whenever it is available) in estimation for the three missing sources, as described in Box1.

| **Box1 Imputing missing disbursements data in the CRS**  Step 1 Obtaining the disbursements difference between DAC2a and CRS at the donor, recipients, and year level (DIF_dry_ = DAC_dry_ -CRS_dry_). The difference is the missed disbursements in CRS for each recipient.  Step 2 For disbursements difference from Source 1 (17 donors not in CRS), we followed the Van de Sijpe’s study and used the sectorshare obtained from the DAC5 for 5 donors that reported to DAC5. For 12 donors that had no information in both CRS and DAC5, we searched through their websites and obtained sector share information from 8 donors (**S5 Table**). For 4 donors (Croatia, Israel, Latvia, Liechtenstein) without any aid information in their websites, we assigned equal share across sectors. We multiplied the sector share to DIF_dry_ and obtained the sector-level DIF_dry_ for each recipient.  Step 3 For disbursements difference from Source 2 (donors reported to both CRS and DAC2 with more years or recipients to DAC2), we used existing CRS disbursements data to obtain a donor’s average share for each sector in each recipient during the period of 2000 and 2014. We multiplied the sector share to DIF_dry_ and obtained the sector-level DIF_dry_ for each recipient.  Step 4 For disbursements difference from Source 3(the largest source of missing before 2012), we used the existing CRS disbursements data to obtain a donor’s share for each sector in each recipient in each year and multiplied the sector share to DIF_dry_ to obtained the sector-level DIF_dry_ for each recipient.  Step 5 Adding the estimated sectorial difference to the CRS disbursements at the donor, recipient, year and sector level (DIF_drys_ + CRS_drys_) and obtaining the disbursement estimates by sectors with imputed values. |
| --- |

(4) Validating imputation methods for missing disbursements data in the CRS

We compared the imputed value of missing disbursements data generated from our method to that from Van de Sijpe’s study by applying the root-mean-square error (RMSE) method. The RMSE measures the difference between imputed values and the observed values and is calculated as the square root of the mean of the squares of the deviations between predicted values and observed values. The smaller the RMSE is, the closer is the imputed value to the actual value, and the more accurate of the imputed value.

To perform the calculation on the RMSE, we used the CRS data 2011 which has the lowest missing rate among all years. We first split the data into two groups: data used for imputation (80% of the existing data), and data used for validation (20% of the existing data). We generated sectoral share using the 80% of the CRS data. We applied two sectoral shares (from the DAC5 and CRS) to the 20% validation data to obtaine imputed values for the 20% of the validation data. We then calculated the difference between the imputed value obtained from each of the methods and actual value for the observations in the 20% validation data (see procedures in **S6 Fig.**).

We obtained the RMSE for each method, which shows that our estimation generates lower RMSE than the Van de Sijpe’s study in aggregated level and in individual sectors except water and sanitation (**S6 Table**). This indicates that our imputation method for recipient-level missing data is better than the Van de Sijpe’s method.

1. Missing rate by sectors

With imputed disbursements difference between CRS and DAC2 at the sector level, we were able to estimate the missing rate by sectors (DIF_drys_ /(DIF_drys_ + CRS_drys_)). The sectoral missing rates between 2000 and 2014 in health, water and sanitation, food and humanitarian assistance, and education are presented in **S7 Fig**. Health sector started with 51% of missing disbursements in 2000 and reduced to 8% or less than since 2005. Education sector had 44% of disbursements data missing in 2000 and reduced to 11% or less since 2006. There was 46% of missing in water and sanitation section in 2000 and 10% or less since 2008. Missing rate in food and humanitarian assistance had the highest missing rate in 2000 (87%) and fell down straightly to 10% in 2006, and went up in 2013 and 2014 due to lack of reporting to the CRS for food aid in the Middle East region from Saudi Arabia. The trends of missing rates suggested similar pattern across sectors and effects of missing data on aid estimates in these sectors are much smaller after 2005.

For the gap between the CRS and DAC in health sector (DIF_dry_), we allocated a proportion of it into RMNCH using available CRS data: we calculated the disbursements share of child and maternal care for each recipient in each year. We then multiplied the share to the DIF_dry_ for each recipient in each year and obtained the imputed disbursements for child and maternal health. We then added the imputed disbursements to the existing CRS disbursements on child and maternal health for each recipient in each year.

1. Allocating regional and unspecified funds to recipients

In both the CRS and DAC data, some donors reported disbursements only at the regional level or labeled it as “Developing countries, unspecified”. Our study on DAC2a disbursements data shows that these unspecified funding has been taking a substantial proportion of total disbursements in recent years (**S8 Fig.**). In 2014, for example, approximately 33% of disbursements in DAC2 had recipients as regions or not specified. While these funds will not affect the global trends analysis, regional or country level estimates could be substantially affected. We followed previous studies^6,7^ and allocated the annual regional or unspecified fund to each recipient based on its proportion in total aid disbursed to the region or to the developing countries in the year using available CRS data.

1. Six sets of disbursements

With various estimation methods, we produced two sets of annual country-level estimates for child survival for each recipient: one derived from non-missing data in CRS (CRS_rys_), and the other including imputed missed disbursements (CRS_rys_+ DIF_rys_). Each set of estimates was calculated in three ways: disbursements to recipients, disbursements to recipients with allocated regional funds, disbursement to recipients with allocated regional and unspecified funds. For each recipient, we will therefore have six sets of estimates for each area, and consider CRS_rys_ as the lower bound and EST_crs_dif_allo_ (CRS_rys_+ DIF_rys_ + regional and unspecified funds allocated to the recipient) as the upper bound of received disbursements. We therefore have six sets of estimates of aid disbursed to health, RMNCH (upper/lower bound), food and humanitarian assistance, water and sanitation, and education in 134 low- and middle-income countries. All disbursements are converted into 2013 US dollars.

**References**

1. OECD (2015) Technical Guide to terms and data in the Creditor Reporting System (CRS) Aid Activities database. <http://www.oecd.org/dac/stats/crsguide.htm> Accessed April 21, 2015
2. 44. <http://apps.who.int/nha/database>

Greco G, Powell-Jackson T, Borghi J, Mills A. (2008) Countdown to 2015: Assessment of Donor Assistance to Maternal, Newborn, and Child Health between 2003 and 2006. Lancet. 371(9620):1268–1275. doi: 10.1016/S0140-6736(08)60561-9. pmid:18406861

Ravishankar N, Gubbins P, Cooley RJ, Leach-Kemon K, Michaud CM, Jamison DT, et al. (2009) Financing global health: tracking development assistance for health from 1990 to 2007. Lancet. 373(9681):2113–2124. doi: 10.1016/S0140-6736(09)60881-3. pmid:19541038

1. Van de Sijpe N. 2012. Is foreign aid fungible? Evidence from the education and health sectors. World Bank Economic Review 27(2), 320-356.
2. Gilbert BJ, Patel V, Farmer PE, Lu C. Assessing Development Assistance for Mental Health in Developing Countries: 2007–2013. PLoS Med 2015;12(6): e1001834. doi:10.1371/journal.pmed.1001834
3. Arregoces L, Daly F, Pitt C, Hsu J, Martinez-Alvarez M, Greco G, Mills A, Berman P, Borghi J. Countdown to 2015: changes in official development assistance to reproductive, maternal, newborn, and child health, and assessment of progress between 2003 and 2012. *Lancet Glob Health* 2015;3: e410–22
