# Supplementary material for: Assessing development assistance for child survival between 2000 and 2014: A multi-sectoral perspective
Source: PLoS One. 2017 Jul 11;12(7):e0178887. doi: 10.1371/journal.pone.0178887 (PMC5507412; doi:10.1371/journal.pone.0178887)
Supplement: S1 Table — (DOCX) [file pone.0178887.s004.docx]

**S1 Table** 134 low- and middle-income recipients in the CRS (according to the income group classification of the World Bank in 2013)

| Low-income countries (51) |
| --- |
| Afghanistan, Angola, Bangladesh, Benin, Bhutan, Burkina Faso, Burundi, Cambodia, Central African Republic, Chad, Comoros, Democratic People's Republic of Korea, Democratic Republic of the Congo, Djibouti, Equatorial Guinea, Eritrea, Ethiopia, Gambia, Guinea, Guinea-Bissau, Haiti, Kenya, Kiribati, Lao People's Democratic Republic, Lesotho, Liberia, Madagascar, Malawi, Mali, Mauritania, Mozambique, Myanmar, Nepal, Niger, Rwanda, Sao Tome and Principe, Senegal, Sierra Leone, Solomon Islands, Somalia, Sudan, Tajikistan, Tanzania, Timor-Leste, Togo, Tuvalu, Uganda, Vanuatu, Yemen, Zambia, Zimbabwe |
| Lower-middle income countries (33) |
| Armenia, Bolivia, Cabo Verde, Cameroon, Congo, Côte d'Ivoire, Egypt, El Salvador, Georgia, Ghana, Guatemala, Guyana, Honduras, India, Indonesia, Kyrgyzstan, Micronesia, Moldova, Mongolia, Morocco, Nicaragua, Nigeria, Pakistan, Papua New Guinea, Paraguay, Philippines, Samoa, Sri Lanka, Swaziland, Syrian Arab Republic, Ukraine, Uzbekistan, Viet Nam. |
| Upper-middle income countries (50) |
| Albania, Algeria, Antigua and Barbuda, Argentina, Azerbaijan, Belarus, Belize, Bosnia and Herzegovina, Botswana, Brazil, Chile, China, Colombia, Costa Rica, Cuba, Dominica, Dominican Republic, Ecuador, Fiji, Former Yugoslav Republic of Macedonia, Gabon, Grenada, Iran, Iraq, Jamaica, Jordan, Kazakhstan, Lebanon, Libya, Malaysia, Maldives, Mauritius, Mexico, Namibia, Palau, Panama, Peru, Saint Lucia, Saint Vincent and the Grenadines, Serbia, Seychelles, South Africa, Suriname, Thailand, Tonga, Tunisia, Turkey, Turkmenistan, Uruguay, Venezuela  13 states were excluded: Cook Islands, Kosovo, Marshall Islands, Montenegro, Montserrat, Nauru, Niue, South Sudan, St. Helena, States Ex-Yugoslavia, Tokelau, Wallis and Futuna, and West Bank and Gaza Strip.) |
