# Supplementary material for: Assessing development assistance for child survival between 2000 and 2014: A multi-sectoral perspective
Source: PLoS One. 2017 Jul 11;12(7):e0178887. doi: 10.1371/journal.pone.0178887 (PMC5507412; doi:10.1371/journal.pone.0178887)
Supplement: S2 Table — (DOCX) [file pone.0178887.s005.docx]

**S2 Table**. Keywords for identifying projects for RMNCH, HIV/AIDS, TB, and health system strengthening

| **Child health**  Baby/babies, boy, child, girl, infant, kid, neonate, newborn, toddler, under age five, acute lower respiratory, acute respiratory infection (ARI), Amoxicillin, ARI prevention, baby friendly hospital initiative (BFHI), birth defect, breastfeed, bronchitis, cerebral palsy, congenital anomaly, cord care, cryptosporidiosis, delayed cord clamping, deworm, diarrhea, diphtheria, Global Alliance for Vaccines and Immunizations (GAVI), growth monitoring, hoxygen therapy, iodine deficiency, international baby food action network (IBFAN), International Council for Control of Iodine Deficiency Disorders (ICCIDD), International Center for Diarrheal Disease Research (ICDDR), Integrated Child Development Services (ICDS), Iodine Deficiency Disorders (IDD) Elimination, International Maternal and Child Health (IMCH), Integrated Management of Childhood Illness (IMCI), immunization, iron supplement, Kangaroo care, Magnesium Sulphate, Maternal, maternal and child health (MCH), Meningitis, measles, MILLENIUM DEVELOPMENT GOAL (MDG) 4, meningitis, maternal, neonatal care, Newborn and Child Health (MNCH), nursery, nurture, orphan, oral rehydration salt (ORS), Oral rehydration solution (ORS), Oral rehydration therapy (ORT), Orphans and Vulnerable Children (OVC), oxygen system, parasite, parenthood, pediatrics, Pertussis, Pneumonia, polio, premature, preprimary preschool, preterm, prevention of mother-to-child transmission (PMTCT), Reproductive, Maternal, Newborn, Child Health (RMNCH), respiratory, rotavirus, Shigella, skin to skin care, stillbirth, stunting, tetanus, therapeutic hypothermia, thermal care, topical emollient therapy, toxoid, underweight, United Nations Children's Fund (UNICEF), vaccine, vitamin.  Projects funded by GAVI and UNICEF.  **Maternal health**  Abortion, antenatal care (ANC), assisted birth, assisted delivery, birth delivery, delivery room, delivery table, safe delivery, sanitary delivery, skilled delivery, skilled-birth delivery, Basic Emergency Obstetric Care (BEmOC), cervical, Comprehensive Emergency Obstetric Care (CEmOC), embryo, embolism haemorrhage, eclampsia, fetal, fetus, female genital mutilation (FGM), Fistula, folic acid, gestation, Gynecology, incubation, induction of labor, International Maternal and Child Health (IMCH), intermittent preventative treatment for malaria in pregnancy (IPTp), intrauterine, labor, maternal, Maternal and Child Health (MCH), midwifery, mother, MILLENIUM DEVELOPMENT GOAL (MDG) 5, Maternal, Newborn and Child Health (MNCH), obstetrics, obstructed labor, placenta, prevention of mother-to-child transmission (PMTCT), perinatal care, postnatal care, postpartum, pregnancy, prenatal care.  **Reproductive health**  Condom, contraception, gender equality and reproductive right, family planning (FP), family size, female genital mutilation (FGM), fertility, injectable, International Planned Parenthood Federation (IPPF), intrauterine device (IUD), microbicide, reproductive health, sepsis, sexual and reproductive health (SRH), utero, United Nations Population Fund (UNFPA), vaginal vasectomy.  Projects funded by United Nations Population Fund (UNFPA).  Projects with purpose name “Family planning” and “Reproductive health care”.  **Nutrition**  Anemia, calcium, dairy, diet, feed, food, growth monitoring, iodine, iron, malnutrition screening, milk, minerals, nourish, nutrition, nutrition, vitamin, zinc.  Projects with purpose name “Basic nutrition”.  **Malaria** Artemisinin, Artemisinin-based Combination Therapy (ACTs), bed-nets, chloroquine, Doxycyclin, Falciparum, Insecticide-treated bed nets (ITNs), Indoor Residual Spraying (IRS),  long lasting insecticide treated nets (LLIN), Lumefantrin, malaria, Mefloquin, mosquito, plasmodium, pyrimethamin, quinin, sulfadoxin. Projects with purpose name “Malaria control”.  **HIV/AIDS**  Abstinence, AIDS, antiretroviral (ARV) therapy (ART), behavior change communication (BCC), Global Fund to Fight AIDS, Tuberculosis, and Malaria (GFATM), HIV, HIV/AIDS, Highly active antiretroviral therapy (HAART), human immunodeficiency virus, microbicide, Millenuim Development Goal 6 (MDG 6), Mother to Child Transmission, prevention of mother-to-child transmission (PMTCT), People Living with HIV/AIDS (PLHA), risky sexual behavior, Testing and Counseling, UNAIDS, Voluntary Counselling and Testing (VCT)  Projects funded with purpose name “STD control including hiv/aids”.  Projects funded by Joint United Nations Programme on HIV/AIDS (UNAIDS).  **TB**  Bacille Calmette-Guerin (BCG), chest X-Ray, Directly Observed Treatment Short course (DOTS), Directly Observed Treatment Short course, Isoniazid, Rifampin, Smear, Sputum, Tubercolosis.  Projects with purpose name “Tuberculosis control”.  **Health system strengthening**  Projects with the following purpose names are categorized as for health system strengthening: (1) “Basic health infrastructure”, (2) “Health personnel development”, (3) “Health policy & administrative management”, (4) “Medical education/training”, (5) “Medical research”, and (6) “Personnel development for population & reproductive health”, and (7) “Population policy and administrative management”.  **Preventing and treating diarrhea, pneumonia**  Acute lower respiratory, Amoxicillin, Acute respiratory infection (ARI) prevention, bronchitis, diarrhea, Integrated Management of Childhood Illness (IMCI), International Center for Diarrhoeal Disease Research (ICDDR), magnesium sulphate, oral rehydration salt (ORS), Oral rehydration solution (ORS), Oral rehydration therapy (ORT), pneumonia, respiratory, Shigella, Zinc.  **Neonatal health**  Baby Friendly Hospital Initiave (BFHI), birth defect, breastfeed, cord care, cerebral palsy, Kangaroo care, low birth weight, newborn, neonatal, natal nursing premature, perterm, prevention of mother to child transmission (PMTCT).  **Vaccine**  Diphtheria, immunization, measle, Pertussis, polio, rotavirus, tetanus toxoid, vaccine. Projects funded by GAVI and International Finance Facility for Immunization (IFFIM). |
| --- |
