# Supplementary material for: Assessing development assistance for child survival between 2000 and 2014: A multi-sectoral perspective
Source: PLoS One. 2017 Jul 11;12(7):e0178887. doi: 10.1371/journal.pone.0178887 (PMC5507412; doi:10.1371/journal.pone.0178887)
Supplement: S3 Table — (DOCX) [file pone.0178887.s006.docx]

**S3 Table** Donors reported to DAC2 and CRS and their available years

| **Donor name** | **Year available in CRS** | **Year available in DAC** |
| --- | --- | --- |
| *Bilateral donors* | | |
| Australia | 2000-2014 | 2000-2014 |
| Austria | 2000-2014 | 2000-2014 |
| Belgium | 2000-2014 | 2000-2014 |
| Bulgaria |  | 2010-2014 |
| Canada | 2000-2014 | 2000-2014 |
| Chinese Taipei |  | 2004-2014 |
| Croatia |  | 2012-2013 |
| Cyprus |  | 2005-2013 |
| Czech Republic (joined in May 2013) | 2011-2014 | 2000-2014 |
| Denmark | 2000-2014 | 2000-2014 |
| Estonia | 2013-2014 | 2000-2014 |
| Finland | 2000-2014 | 2000-2014 |
| France | 2000-2014 | 2000-2014 |
| Germany | 2000-2014 | 2000-2014 |
| Greece | 2002-2014 | 2000-2014 |
| Hungary | 2014 only | 2003-2014 |
| Iceland | 2011-2014 | 2000-2014 |
| Ireland | 2000-2014 | 2000-2014 |
| Italy | 2000-2014 | 2000-2014 |
| Japan | 2000-2014 | 2000-2014 |
| Kazakhstan | 2013-2014 | 2013-2014 |
| Korea | 2002, 2003, 2006-2014 | 2000-2014 |
| Israel |  | 2000-2014 |
| Latvia |  | 2002-2014 |
| Liechtenstein |  | 2007-2014 |
| Lithuania | 2014 only | 2001-2014 |
| Luxembourg (joined in 2000) | 2001-2014 | 2000-2014 |
| Malta |  | 2009-2014 |
| Netherlands | 2000-2014 | 2000-2014 |
| New Zealand | 2002-2014 | 2000-2014 |
| Norway | 2000-2014 | 2000-2014 |
| Poland | 2013-2014 | 2000-2014 |
| Portugal | 2000-2014 | 2000-2014 |
| Romania | 2014 only | 2008-2014 |
| Russia |  | 2010-2014 |
| Saudi Arabia |  | 2000-2014 |
| Slovak Republic | 2013-2014 | 2000-2014 |
| Slovenia | 2010-2014 | 2005-2014 |
| Spain | 2000-2014 | 2000-2014 |
| Sweden | 2000-2014 | 2000-2014 |
| Switzerland | 2000-2014 | 2000-2014 |
| Thailand |  | 2006-2014 |
| Turkey |  | 2000-2014 |
| United Kingdom | 2000-2014 | 2000-2014 |
| United States | 2000-2014 | 2000-2014 |
| Kuwait (KFAED) | 2010 - 2014 | 2000-2014 |
| United Arab Emirates | 2009-2014 | 2000-2014 |
| *Multilateral donors* | | |
| Adaptation Fund | 2010-2014 | 2013-2014 |
| African Development Bank | 2001-2014 | 2001-2014 |
| African Development Fund | 2000-2014 | 2000-2014 |
| Arab Fund | 2008-2014 | 2008-2014 |
| Asian Development Bank, Special Fund | 2000-2014 | 2000-2014 |
| Arab Bank for Economic Development in Africa | 2011-2014 | 2000-2001, 2004-2014 |
| Caribbean Development Bank |  | 2000-2014 |
| Climate Investment Funds | 2012-2014 | 2013-2014 |
| Council of Europe Development Bank | 2010-2014 | 2010-2014 |
| European Bank for Reconstruction and Development [EBRD] |  | 2000-2008 |
| European Union Institution | 2000-2014 | 2000-2014 |
| Food and Agriculture Organisation | 2013 |  |
| Global Alliance for Vaccines and Immunisation | 2001-2014 | 2001-2014 |
| Global Environment Facility | 2000-2014 | 2001-2014 |
| Global Fund to Fight AIDS, Tuberculosis and Malaria | 2002-2014 | 2002-2014 |
| Global Green Growth Institute | 2013-2014 | 2013-2014 |
| Inter-American Development Bank, Special Operation Fund | 2000-2014 | 2000-2014 |
| International Atomic Energy Agency |  | 2006-2014 |
| International Fund for Agricultural Development | 2000-2014 | 2000-2014 |
| International Monetary Fund (Concessional Trust Funds) | 2000-2014 | 2000-2014 |
| International Development Association (IDA, World Bank) | 2000-2014 | 2000-2014 |
| Islāmic Development Bank | 2000-2014 | 2000-2014 |
| Montreal Protocol |  | 2000-2014 |
| Nordic Development Fund | 2009-2014 | 2000-2014 |
| OPEC Fund for International Development | 2009-2014 | 2000-2014 |
| Organization for Security and Co-operation in Europe | 2010-2014 | 2010-2014 |
| United Nations Peacebuilding Fund | 2007-2014 | 2007-2014 |
| Joint United Nations Programme on HIV/AIDS | 2001-2014 | 2005-2014 |
| United Nations Development Programme (UNDP) | 2004-2014 | 2000-2014 |
| United Nations Economic Commission for Europe (UNECE) | 2008-2014 | 2008-2014 |
| United Nations Population Fund | 2001-2014 | 2000-2014 |
| United Nations regular program me for technical assistance |  | 2000-2008 |
| United Nations Refugee Agency | 2011-2014 | 2000-2014 |
| United Nations Children’s Fund | 2000-2014 | 2000-2014 |
| United Nations Relief and Works Agency | 2005-2014 | 2000-2014 |
| World Food Programme (WFP) | 2008-2014 | 2000-2014 |
| World Health Organization (WHO) | 2009-2014 | 2009-2014 |
| *Private donors* | | |
| Bill & Melinda Gates Foundation | 2009-2014 | 2009-2014 |
