# Supplementary material for: Assessing development assistance for child survival between 2000 and 2014: A multi-sectoral perspective
Source: PLoS One. 2017 Jul 11;12(7):e0178887. doi: 10.1371/journal.pone.0178887 (PMC5507412; doi:10.1371/journal.pone.0178887)
Supplement: S4 Table — (DOCX) [file pone.0178887.s007.docx]

**S4 Table**. Websites and aid information for 8 donors

| **Donor name** | **Website** | **Year of data available** | **Access date** |
| --- | --- | --- | --- |
| Chinese Taipei | <http://www.mofa.gov.tw/Upload/RelFile/17/262/8a4267f8-9d77-44f3-8dfc-f2ecdc0f38dc.pdf> | 2014 | November 14th, 2015 |
|  | http://www.mofa.gov.tw/Upload/RelFile/17/262/cb6707b1-a9d4-49a1-a162-f42b71c50e68.pdf | 2013 | November 14th, 2015 |
| Russia | <http://www.alnap.org/pool/files/dp-russia-humanitarian-donor-150713-en.pdf> | 2006-2013 | September 10th, 2014 |
| Cyprus | http://www.cyprusaid.gov.cy/planning/cyprusaid.nsf/all/DAB6D9BBCC7DEF8DC2257C36003CD833/$file/2012%20-%20ENGLISH.pdf?openelement | 2012 | November 14th, 2015 |
| Thailand | http://www.th.undp.org/content/dam/thailand/docs/ThailandODAReport2007-08.pdf | 2007 | November 14th, 2015 |
| International Atomic Energy Agency (IAEA) | https://www.iaea.org/sites/default/files/gc59-7_en.pdf | 2014 | November 14th, 2015 |
|  | http://www.iaea.org/Publications/Reports/Anrep2013/anrep2013_full.pdf | 2013 | November 14th, 2015 |
| Saudi Arabia | https://docs.google.com/spreadsheet/ccc?key=0AvGCmVxSBN08dGI1bWx5LVdocTBVSmY4NVViV3NNdmc#gid=2 | 2009-2013 | November 14th, 2015 |
|  | https://docs.google.com/spreadsheet/ccc?key=0AvGCmVxSBN08dGI1bWx5LVdocTBVSmY4NVViV3NNdmc#gid=2 | 2006-2011 | May 6th, 2014 |
| United Nations regular programme for technical assistance (UNTA) | http://www.un.org/ga/search/view_doc.asp?symbol=A/70/6(Sect.23) | 2012-2015 | November 14th, 2015 |
|  | http://www.un.org/ga/search/view_doc.asp?symbol=A/68/6(Sect.23) | 2010-2013 | November 14th, 2015 |
|  | http://www.un.org/ga/search/view_doc.asp?symbol=A/66/6(Sect.23) | 2008-2011 | November 14th, 2015 |
|  | http://www.un.org/ga/search/view_doc.asp?symbol=A/64/6(Sect.22) | 2006-2009 | November 14th, 2015 |
|  | http://www.un.org/Docs/journal/asp/ws.asp?m=A/62/6(Sect.22) | 2004-2007 | November 14th, 2015 |
| Montreal Protocol* | <http://ozone.unep.org/en/treaties-and-decisions/montreal-protocol-substances-deplete-ozone-layer> |  | May 6th, 2014 |

*The Montreal Protocol on Substances that Deplete the Ozone Layer was designed to reduce the production and consumption of ozone depleting substance. We therefore assigned its funds to environment protection sector.
