# Supplementary material for: Assessing development assistance for child survival between 2000 and 2014: A multi-sectoral perspective
Source: PLoS One. 2017 Jul 11;12(7):e0178887. doi: 10.1371/journal.pone.0178887 (PMC5507412; doi:10.1371/journal.pone.0178887)
Supplement: S5 Table — (DOCX) [file pone.0178887.s008.docx]

**S5 Table** RMSE from the two methods

|  | Lu’s et al. | Van de Sijpe |
| --- | --- | --- |
| Sector share | | |
| Health | 0·061 | 0·075 |
| Education | 0·056 | 0·061 |
| Water/Sanitation | 0·074 | 0·077 |
| Food and Food Security | 0·085 | 0·173 |
| Disaster response and relief | 0·089 | 0·16 |
| Disbursements at donor, recipient, and year level | | |
| Health | 6·33 | 8·78 |
| Education | 2·81 | 2·91 |
| Water/Sanitation | 4·37 | 4·53 |
| Food and Food Security | 5·47 | 6·88 |
| Disaster response and relief | 3·52 | 6·42 |
