# Supplementary material for: Assessing development assistance for child survival between 2000 and 2014: A multi-sectoral perspective
Source: PLoS One. 2017 Jul 11;12(7):e0178887. doi: 10.1371/journal.pone.0178887 (PMC5507412; doi:10.1371/journal.pone.0178887)
Supplement: S6 Table — (DOCX) [file pone.0178887.s009.docx]

**S6 Table.** Countdown priority countries

| 74 Countdown priority states  Afghanistan, Angola, Azerbaijan, Bangladesh, Benin, Bolivia, Botswana, Brazil, Burkina Faso, Burundi, Cambodia, Cameroon, Central African Republic, Chad, China, Comoros, Congo, Cote d'Ivoire, Democratic People's Republic of Korea, Democratic Republic of the Congo, Djibouti, Egypt, Equatorial Guinea, Eritrea, Ethiopia, Gabon, Gambia, Ghana, Guatemala, Guinea, Guinea-Bissau, Haiti, India, Indonesia, Iraq, Kenya, Kyrgyzstan, Lao People's Democratic Republic, Lesotho, Liberia, Madagascar, Malawi, Mali, Mauritania, Mexico, Morocco, Mozambique, Myanmar, Nepal, Niger, Nigeria, Pakistan, Papua New Guinea, Peru, Philippines, Rwanda, Sao Tome and Principe, Senegal, Sierra Leone, Solomon Islands, Somalia, South Africa, Sudan, Swaziland, Tajikistan, Tanzania, Togo, Turkmenistan, Uganda, Uzbekistan, Viet Nam, Yemen, Zambia, Zimbabwe  67 Countdown states with high child mortality rate in 2000  Afghanistan, Angola, Azerbaijan, Bangladesh, Benin, Bolivia, Botswana, Burkina Faso, Burundi, Cambodia, Cameroon, Central African Republic, Chad, Comoros, Congo, Cote d'Ivoire, Democratic People's Republic of Korea, Democratic Republic of the Congo, Djibouti, Egypt, Equatorial Guinea, Eritrea, Ethiopia, Gabon, Gambia, Ghana, Guatemala, Guinea, Guinea-Bissau, Haiti, India, Indonesia, Iraq, Kenya, Kyrgyzstan, Lao People's Democratic Republic, Lesotho, Liberia, Madagascar, Malawi, Mali, Mauritania, Mozambique, Morocco, Myanmar, Nepal, Niger, Nigeria, Pakistan, Papua New Guinea, Rwanda, Sao Tome and Principe, Senegal, Sierra Leone, Somalia, South Africa, Sudan, Swaziland, Tajikistan, Tanzania, Togo, Turkmenistan, Uganda, Uzbekistan, Yemen, Zambia, Zimbabwe  42 Countdown states with high reliance on health aid  Afghanistan, Benin, Burkina Faso, Burundi, Cambodia, Central African Republic, Chad, Comoros, Democratic Republic of the Congo, Djibouti, Eritrea, Ethiopia, Gambia, Ghana, Guinea, Guinea-Bissau, Haiti, Kenya, Kyrgyzstan, Lao People's Democratic Republic, Lesotho, Liberia, Madagascar, Malawi, Mali, Mauritania, Mozambique, Nepal, Niger, Nigeria, Papua New Guinea, Rwanda, Sao Tome and Principe, Senegal, Sierra Leone, Somalia, Solomon Island, Tanzania, Togo, Uganda, Zambia, Zimbabwe  25 Countdown states in fragile status  We used the Fragile States Index from the Fund for Peace (<http://global.fundforpeace.org/cewa#fsi>) and identified the top 20 countries with the highest fragile index each year. 25 countries were categorized as fragile states.  Afghanistan, Burundi, Central African Republic, Chad, Côte d'Ivoire, Democratic People's Republic of Korea, Democratic Republic of the Congo, Ethiopia, Guinea, Guinea-Bissau, Haiti, Iraq, Kenya, Liberia, Myanmar, Nepal, Niger, Nigeria, Pakistan, Sierra Leone, Somalia, Sudan, Uganda, Yemen, Zimbabwe  15 Countdown states meeting MDG 4 and highly relied on health aid  Countries with more than 10% of the total health expenditure from development health assistance for 10 year or more is defined as countries with high-level of reliance on health aid. We chose this cutoff point based on government health spending as % of total government spending: the median is about 10% among the 134 countries.  Cambodia, Eritrea, Ethiopia, Kyrgyzstan, Liberia, Madagascar, Malawi, Mozambique, Nepal, Niger, Rwanda, Senegal, Tanzania, Uganda, Zambia |
| --- |
