# Supplementary material for: Assessing development assistance for child survival between 2000 and 2014: A multi-sectoral perspective
Source: PLoS One. 2017 Jul 11;12(7):e0178887. doi: 10.1371/journal.pone.0178887 (PMC5507412; doi:10.1371/journal.pone.0178887)
Supplement: S7 Table — (DOCX) [file pone.0178887.s010.docx]

**S7 Table** Top 10 states receiving the largest amount of aid (upper-bound) over 15 years: total vs. per capita

1. Total amount over 15 years in 2013 USD

| Health (40%) | | RMNCH (48%) | | Food/Humanitarian Assistance (48%) | | Water/Sanitation (35%) | | Education (42%) | |
| --- | --- | --- | --- | --- | --- | --- | --- | --- | --- |
| Country | Total15yrs | Country | Total_15yrs | Country | Total_15yrs | Country | Total_15yrs | Country | Total_15yrs |
| India | 1.75E+10 | India | 9.10E+09 | Sudan | 1.71E+10 | India | 4.35E+09 | India | 6.86E+09 |
| Nigeria | 1.33E+10 | Nigeria | 6.07E+09 | Afghanistan | 1.56E+10 | China | 4.28E+09 | Bangladesh | 4.77E+09 |
| Ethiopia | 1.09E+10 | Ethiopia | 4.62E+09 | Ethiopia | 1.35E+10 | Viet Nam | 3.77E+09 | Pakistan | 2.40E+09 |
| Tanzania | 1.06E+10 | Bangladesh | 4.00E+09 | Iraq | 1.01E+10 | Iraq | 3.26E+09 | Afghanistan | 2.26E+09 |
| Kenya | 9.89E+09 | Pakistan | 3.91E+09 | Pakistan | 1.01E+10 | Morocco | 2.35E+09 | Tanzania | 2.00E+09 |
| Uganda | 8.45E+09 | Tanzania | 3.39E+09 | Syrian Arab Republic | 8.88E+09 | Tanzania | 2.23E+09 | Jordan | 1.96E+09 |
| South Africa | 8.40E+09 | Kenya | 3.32E+09 | Democratic Republic of the Congo | 8.09E+09 | Bangladesh | 1.91E+09 | Indonesia | 1.71E+09 |
| Afghanistan | 7.85E+09 | Democratic Republic of the Congo | 3.06E+09 | Somalia | 6.65E+09 | Jordan | 1.90E+09 | Ethiopia | 1.64E+09 |
| Mozambique | 7.81E+09 | Afghanistan | 2.70E+09 | Haiti | 5.26E+09 | Indonesia | 1.76E+09 | Uganda | 1.46E+09 |
| Pakistan | 6.74E+09 | Uganda | 2.39E+09 | Kenya | 5.12E+09 | Ethiopia | 1.69E+09 | Viet Nam | 1.42E+09 |

1. Per capita over 15 years in 2013 USD

| Health | | RMNCH | | Food/Human. Assis. | | Water/Sanitation | | Education | |
| --- | --- | --- | --- | --- | --- | --- | --- | --- | --- |
| Country | Per capita | Country | Per capita | Country | Per capita | Country | Per capita | Country | Per capita |
| Micronesia | 229.30 | Tuvalu | 20.24 | Tuvalu | 178.40 | Tuvalu | 68.61 | Tuvalu | 86.454 |
| Tuvalu | 145.46 | Sao Tome and Principe | 18.38 | Grenada | 59.19 | Samoa | 43.44 | Micronesia | 52.827 |
| Tonga | 76.48 | Liberia | 13.00 | Lebanon | 57.87 | Palau | 35.09 | Tonga | 40.856 |
| Sao Tome and Principe | 59.76 | Timor-Leste | 11.64 | Somalia | 49.67 | Tonga | 31.77 | Vanuatu | 39.889 |
| Solomon Islands | 56.77 | Micronesia | 11.59 | Maldives | 49.04 | Dominica | 25.99 | Samoa | 37.602 |
| Namibia | 53.35 | Gambia | 10.18 | Kyrgyzstan | 47.07 | Kiribati | 23.42 | Kiribati | 37.262 |
| Kiribati | 51.52 | Malawi | 9.31 | Palau | 45.35 | Cabo Verde | 22.55 | Grenada | 29.111 |
| Botswana | 50.92 | Sierra Leone | 8.99 | Jordan | 44.76 | Jordan | 22.32 | Solomon Islands | 26.419 |
| Vanuatu | 50.47 | Zambia | 8.16 | Liberia | 40.85 | Guyana | 21.63 | Jordan | 23.024 |
| Palau | 49.65 | Solomon Islands | 8.06 | Afghanistan | 40.44 | Sao Tome and Principe | 20.17 | Palau | 22.487 |
