# Supplementary material for: Assessing development assistance for child survival between 2000 and 2014: A multi-sectoral perspective
Source: PLoS One. 2017 Jul 11;12(7):e0178887. doi: 10.1371/journal.pone.0178887 (PMC5507412; doi:10.1371/journal.pone.0178887)
Supplement: S8 Table — (DOCX) [file pone.0178887.s011.docx]

**S8 Table** Upper-bound per capita disbursements to each sector of child survival in 74 Countdown countries, 2000-2014

1. *RMNCH*

|  | **2000** | **2001** | **2002** | **2003** | **2004** | **2005** | **2006** | **2007** | **2008** | **2009** | **2010** | **2011** | **2012** | **2013** | **2014** |
| --- | --- | --- | --- | --- | --- | --- | --- | --- | --- | --- | --- | --- | --- | --- | --- |
| **Afghanistan** | 1.23 | 2.61 | 4.33 | 3.98 | 1.20 | 1.61 | 2.21 | 9.26 | 5.87 | 11.29 | 10.44 | 11.01 | 9.77 | 10.25 | 12.73 |
| **Angola** | 2.14 | 3.26 | 3.13 | 1.56 | 0.90 | 4.15 | 2.33 | 4.41 | 4.57 | 4.26 | 4.66 | 3.03 | 4.71 | 4.44 | 3.77 |
| **Azerbaijan** | 0.88 | 0.69 | 0.38 | 0.28 | 0.38 | 0.71 | 0.47 | 0.63 | 1.19 | 1.67 | 2.40 | 2.01 | 1.02 | 1.97 | 1.28 |
| **Bangladesh** | 1.42 | 1.51 | 1.33 | 1.54 | 1.05 | 1.62 | 2.38 | 1.07 | 1.53 | 2.31 | 2.26 | 2.13 | 1.68 | 2.88 | 2.40 |
| **Benin** | 1.89 | 2.31 | 1.56 | 1.88 | 1.91 | 2.76 | 1.99 | 4.86 | 5.60 | 7.63 | 8.48 | 8.60 | 6.93 | 8.65 | 9.56 |
| **Bolivia** | 4.93 | 6.98 | 2.32 | 3.67 | 2.00 | 1.74 | 2.23 | 3.94 | 3.92 | 4.34 | 5.72 | 5.90 | 5.64 | 4.41 | 2.53 |
| **Botswana** | 2.64 | 1.32 | 1.90 | 2.67 | 0.91 | 1.69 | 0.69 | 2.38 | 0.58 | 0.92 | 0.58 | 2.21 | 3.51 | 3.33 | 3.51 |
| **Brazil** | 0.00 | 0.04 | 0.06 | 0.01 | 0.02 | 0.02 | 0.01 | 0.02 | 0.02 | 0.07 | 0.07 | 0.08 | 0.03 | 0.03 | 0.04 |
| **Burkina Faso** | 3.34 | 2.19 | 2.03 | 1.68 | 1.83 | 4.38 | 2.46 | 3.55 | 3.64 | 6.68 | 8.43 | 3.95 | 8.61 | 8.47 | 8.23 |
| **Burundi** | 0.93 | 1.25 | 2.15 | 3.45 | 2.70 | 3.21 | 3.39 | 4.51 | 5.07 | 5.38 | 6.69 | 5.55 | 6.21 | 8.37 | 8.53 |
| **Cambodia** | 1.01 | 2.37 | 2.50 | 3.04 | 1.88 | 5.85 | 5.10 | 6.68 | 3.62 | 5.83 | 10.07 | 10.81 | 6.24 | 7.22 | 10.24 |
| **Cameroon** | 0.56 | 1.53 | 1.20 | 1.12 | 1.88 | 2.25 | 2.60 | 3.96 | 1.66 | 2.91 | 1.76 | 6.96 | 3.06 | 4.40 | 3.70 |
| **Central African Republic** | 1.11 | 1.04 | 3.22 | 1.32 | 1.66 | 2.07 | 3.48 | 2.98 | 5.83 | 3.02 | 4.99 | 5.25 | 5.03 | 7.94 | 8.55 |
| **Chad** | 2.39 | 2.42 | 1.90 | 1.50 | 1.93 | 2.30 | 1.44 | 2.24 | 2.06 | 2.33 | 5.34 | 3.01 | 2.42 | 6.40 | 5.14 |
| **China** | 0.07 | 0.04 | 0.05 | 0.05 | 0.03 | 0.04 | 0.03 | 0.05 | 0.02 | 0.04 | 0.08 | 0.03 | 0.04 | 0.01 | 0.01 |
| **Comoros** | 2.40 | 3.47 | 5.80 | 7.60 | 6.21 | 3.14 | 3.84 | 3.00 | 4.84 | 2.51 | 11.75 | 5.77 | 10.79 | 10.05 | 5.59 |
| **Congo** | 1.65 | 2.70 | 1.30 | 1.00 | 0.77 | 0.83 | 2.47 | 4.13 | 2.10 | 3.05 | 10.70 | 6.07 | 6.37 | 6.32 | 2.08 |
| **Cote d'Ivoire** | 0.73 | 0.99 | 1.05 | 1.69 | 0.81 | 0.85 | 0.71 | 1.39 | 1.48 | 2.84 | 5.49 | 2.71 | 3.28 | 5.80 | 6.79 |
| **Democratic People's Republic of Korea** | 0.19 | 0.10 | 0.20 | 0.29 | 0.29 | 0.48 | 0.32 | 0.37 | 0.24 | 0.90 | 1.03 | 0.61 | 0.77 | 0.97 | 1.05 |
| **Democratic Republic of the Congo** | 0.50 | 0.89 | 0.86 | 1.02 | 1.09 | 1.54 | 1.54 | 2.13 | 3.76 | 4.06 | 4.54 | 4.76 | 6.63 | 6.07 | 6.82 |
| **Djibouti** | 1.54 | 5.38 | 6.57 | 4.31 | 6.69 | 7.77 | 6.81 | 18.30 | 6.23 | 9.50 | 4.47 | 3.25 | 7.00 | 5.72 | 5.89 |
| **Egypt** | 0.93 | 0.90 | 1.49 | 0.72 | 0.91 | 0.63 | 0.51 | 0.58 | 0.58 | 0.49 | 0.29 | 0.14 | 0.22 | 0.20 | 0.30 |
| **Equatorial Guinea** | 9.13 | 3.29 | 3.80 | 3.06 | 2.26 | 3.05 | 9.96 | 5.95 | 17.30 | 14.40 | 19.17 | 15.28 | 2.42 | 0.32 | 1.15 |
| **Eritrea** | 2.22 | 5.06 | 5.93 | 8.13 | 7.65 | 9.84 | 3.07 | 6.71 | 5.62 | 3.46 | 10.48 | 3.15 | 3.34 | 5.32 | 8.00 |
| **Ethiopia** | 1.05 | 1.25 | 1.10 | 1.51 | 1.03 | 1.36 | 4.48 | 6.02 | 2.98 | 5.18 | 3.92 | 5.32 | 5.33 | 7.21 | 5.87 |
| **Gabon** | 1.05 | 1.96 | 1.75 | 1.02 | 2.19 | 4.82 | 5.46 | 4.10 | 2.03 | 4.46 | 1.44 | 0.78 | 1.22 | 1.27 | 1.66 |
| **Gambia** | 3.39 | 2.50 | 4.89 | 4.88 | 5.75 | 9.15 | 3.84 | 23.56 | 6.61 | 10.56 | 11.36 | 10.54 | 9.48 | 21.33 | 16.26 |
| **Ghana** | 0.33 | 3.13 | 1.50 | 1.43 | 1.88 | 2.90 | 2.18 | 3.60 | 2.82 | 6.86 | 7.79 | 5.86 | 8.53 | 9.29 | 7.70 |
| **Guatemala** | 0.32 | 0.44 | 3.45 | 0.73 | 1.34 | 1.69 | 2.53 | 2.74 | 3.01 | 4.26 | 2.97 | 4.37 | 3.51 | 3.84 | 4.60 |
| **Guinea** | 3.48 | 4.90 | 3.77 | 1.12 | 1.14 | 2.89 | 2.94 | 2.28 | 2.15 | 2.92 | 4.92 | 3.18 | 6.93 | 4.21 | 8.46 |
| **Guinea-Bissau** | 1.06 | 1.60 | 2.32 | 2.43 | 2.02 | 3.73 | 1.98 | 6.89 | 5.41 | 4.61 | 14.58 | 9.97 | 12.92 | 26.43 | 5.56 |
| **Haiti** | 4.49 | 3.51 | 1.84 | 1.46 | 1.45 | 4.41 | 5.70 | 10.31 | 6.73 | 4.72 | 5.87 | 8.78 | 9.17 | 9.88 | 7.95 |
| **India** | 0.46 | 0.51 | 0.66 | 0.35 | 0.42 | 0.50 | 0.37 | 0.53 | 0.43 | 0.52 | 0.70 | 0.71 | 0.60 | 0.46 | 0.47 |
| **Indonesia** | 0.16 | 0.28 | 0.48 | 0.29 | 0.38 | 0.32 | 0.26 | 0.24 | 0.39 | 0.51 | 0.58 | 0.39 | 0.46 | 0.61 | 0.54 |
| **Iraq** | 0.26 | 0.16 | 0.17 | 0.37 | 0.28 | 0.14 | 0.26 | 2.11 | 0.85 | 0.45 | 0.11 | 0.10 | 0.77 | 0.46 | 1.15 |
| **Kenya** | 2.04 | 3.48 | 3.82 | 3.17 | 2.34 | 5.08 | 8.61 | 7.52 | 3.80 | 6.75 | 7.60 | 7.49 | 6.94 | 8.29 | 8.42 |
| **Kyrgyzstan** | 0.44 | 1.25 | 2.72 | 4.05 | 1.18 | 1.51 | 1.12 | 3.56 | 2.90 | 1.76 | 3.02 | 1.89 | 3.83 | 5.52 | 6.36 |
| **Lao People's Democratic Republic** | 1.92 | 3.47 | 5.57 | 3.80 | 2.29 | 3.05 | 3.45 | 4.66 | 5.59 | 4.99 | 4.29 | 4.26 | 5.23 | 7.36 | 9.59 |
| **Lesotho** | 4.05 | 2.50 | 2.99 | 3.39 | 2.32 | 1.19 | 2.48 | 4.28 | 6.55 | 9.38 | 6.02 | 11.40 | 7.67 | 8.92 | 6.25 |
| **Liberia** | 0.48 | 2.48 | 1.49 | 3.01 | 3.51 | 4.21 | 5.89 | 5.28 | 11.27 | 14.94 | 22.44 | 23.73 | 25.68 | 24.17 | 26.83 |
| **Madagascar** | 1.51 | 2.34 | 2.22 | 2.86 | 1.89 | 2.52 | 2.35 | 5.49 | 4.25 | 4.44 | 8.86 | 5.35 | 5.43 | 7.45 | 6.61 |
| **Malawi** | 10.46 | 4.61 | 7.54 | 7.85 | 3.66 | 3.18 | 3.68 | 5.79 | 6.12 | 8.45 | 7.84 | 14.45 | 12.12 | 19.38 | 18.06 |
| **Mali** | 1.75 | 2.52 | 1.96 | 1.96 | 2.04 | 2.29 | 3.82 | 4.42 | 5.01 | 6.32 | 7.79 | 9.71 | 9.92 | 10.90 | 11.32 |
| **Mauritania** | 2.59 | 2.64 | 4.39 | 3.62 | 2.66 | 1.75 | 4.76 | 6.17 | 3.66 | 4.51 | 4.09 | 3.40 | 2.33 | 4.21 | 4.88 |
| **Mexico** | 0.05 | 0.08 | 0.11 | 0.05 | 0.03 | 0.03 | 0.03 | 0.04 | 0.02 | 0.01 | 0.03 | 0.01 | 0.03 | 0.01 | 0.02 |
| **Morocco** | 0.90 | 0.28 | 0.61 | 1.15 | 0.88 | 0.54 | 0.45 | 1.98 | 0.74 | 0.21 | 0.16 | 0.14 | 0.25 | 0.24 | 0.10 |
| **Mozambique** | 2.57 | 3.30 | 4.47 | 3.43 | 2.84 | 2.98 | 3.96 | 5.61 | 4.74 | 4.62 | 6.95 | 7.10 | 7.78 | 8.86 | 11.63 |
| **Myanmar** | 0.26 | 0.35 | 0.42 | 0.41 | 0.40 | 0.57 | 0.53 | 1.08 | 0.47 | 0.68 | 1.08 | 1.09 | 2.26 | 1.70 | 3.43 |
| **Nepal** | 1.36 | 2.22 | 2.62 | 1.06 | 1.01 | 2.29 | 2.81 | 2.06 | 2.44 | 2.53 | 3.45 | 2.62 | 2.96 | 3.21 | 3.65 |
| **Niger** | 1.07 | 1.61 | 1.64 | 1.55 | 1.30 | 2.63 | 3.61 | 4.85 | 5.81 | 5.29 | 6.15 | 4.83 | 3.21 | 5.91 | 7.85 |
| **Nigeria** | 0.90 | 1.05 | 1.15 | 0.97 | 1.12 | 1.06 | 1.43 | 2.63 | 2.14 | 5.22 | 2.51 | 3.71 | 4.13 | 5.46 | 4.91 |
| **Pakistan** | 0.40 | 0.48 | 0.51 | 0.72 | 0.30 | 0.83 | 1.36 | 1.85 | 1.50 | 2.18 | 2.07 | 1.92 | 2.86 | 3.60 | 2.55 |
| **Papua New Guinea** | 3.06 | 5.05 | 3.66 | 4.48 | 4.58 | 1.98 | 2.91 | 2.42 | 4.14 | 9.22 | 2.63 | 6.39 | 6.42 | 6.02 | 4.12 |
| **Peru** | 0.27 | 0.43 | 1.03 | 0.61 | 0.15 | 0.61 | 0.44 | 0.68 | 0.65 | 3.15 | 1.44 | 1.04 | 1.94 | 1.53 | 0.73 |
| **Philippines** | 0.53 | 0.64 | 0.47 | 0.55 | 0.34 | 0.81 | 0.88 | 0.83 | 0.61 | 0.74 | 0.92 | 0.64 | 0.69 | 0.67 | 0.72 |
| **Rwanda** | 3.03 | 5.67 | 2.53 | 2.80 | 3.24 | 4.76 | 10.26 | 4.67 | 9.76 | 14.06 | 7.51 | 10.83 | 11.49 | 10.74 | 11.74 |
| **Sao Tome and Principe** | 2.73 | 13.38 | 10.93 | 10.62 | 7.39 | 19.16 | 15.01 | 20.19 | 25.73 | 9.11 | 14.90 | 30.38 | 10.32 | 43.13 | 32.08 |
| **Senegal** | 1.11 | 1.86 | 1.94 | 2.77 | 3.01 | 3.59 | 5.20 | 4.17 | 6.17 | 7.71 | 6.83 | 6.33 | 9.74 | 9.06 | 13.44 |
| **Sierra Leone** | 1.87 | 1.97 | 3.65 | 2.42 | 1.62 | 2.23 | 3.30 | 6.91 | 6.12 | 12.82 | 19.10 | 13.67 | 12.57 | 16.45 | 19.18 |
| **Solomon Islands** | 1.18 | 1.60 | 1.81 | 1.14 | 2.65 | 2.38 | 0.82 | 1.79 | 21.25 | 24.31 | 18.82 | 10.02 | 8.77 | 14.10 | 3.78 |
| **Somalia** | 2.08 | 4.10 | 2.08 | 1.05 | 2.06 | 2.33 | 1.66 | 3.49 | 1.82 | 3.66 | 4.46 | 6.09 | 10.64 | 9.38 | 11.86 |
| **South Africa** | 0.87 | 0.86 | 0.24 | 1.00 | 0.57 | 0.87 | 0.21 | 0.77 | 0.75 | 0.35 | 1.01 | 1.03 | 0.99 | 1.31 | 1.23 |
| **Sudan** | 0.92 | 0.85 | 0.92 | 0.81 | 1.18 | 2.95 | 1.94 | 3.58 | 3.65 | 3.53 | 5.13 | 3.18 | 4.76 | 3.85 | 3.60 |
| **Swaziland** | 1.15 | 1.28 | 2.85 | 13.34 | 7.29 | 22.96 | 1.56 | 12.31 | 12.45 | 6.31 | 4.02 | 7.03 | 8.22 | 7.28 | 8.95 |
| **Tajikistan** | 0.56 | 2.02 | 0.94 | 0.57 | 0.32 | 0.88 | 0.85 | 1.28 | 2.85 | 1.71 | 4.15 | 2.43 | 3.99 | 3.06 | 3.26 |
| **Tanzania** | 2.90 | 2.26 | 1.90 | 1.16 | 1.44 | 3.67 | 3.60 | 5.37 | 7.38 | 6.95 | 7.32 | 6.54 | 7.59 | 8.86 | 8.85 |
| **Togo** | 1.20 | 1.02 | 1.08 | 1.26 | 1.51 | 2.05 | 0.63 | 2.37 | 3.35 | 3.28 | 4.25 | 7.29 | 1.42 | 7.16 | 4.87 |
| **Turkmenistan** | 4.09 | 8.30 | 1.59 | 0.76 | 1.80 | 0.56 | 1.35 | 0.69 | 0.40 | 0.39 | 0.37 | 0.32 | 0.39 | 0.28 | 0.31 |
| **Uganda** | 3.65 | 3.64 | 3.32 | 3.38 | 2.30 | 5.43 | 5.24 | 5.25 | 2.89 | 4.66 | 4.78 | 5.37 | 9.66 | 7.13 | 8.87 |
| **Uzbekistan** | 0.42 | 0.65 | 0.57 | 0.21 | 0.16 | 0.28 | 0.32 | 0.47 | 0.53 | 0.92 | 0.71 | 0.50 | 0.34 | 0.38 | 1.66 |
| **Viet Nam** | 0.51 | 0.71 | 0.74 | 0.63 | 0.53 | 0.68 | 0.55 | 0.78 | 0.66 | 0.60 | 0.73 | 1.00 | 0.64 | 1.18 | 0.84 |
| **Yemen** | 2.88 | 2.18 | 3.04 | 3.00 | 1.22 | 2.57 | 2.25 | 2.83 | 2.53 | 2.25 | 2.93 | 2.93 | 4.22 | 6.11 | 7.85 |
| **Zambia** | 3.41 | 1.80 | 5.91 | 4.74 | 4.83 | 5.25 | 3.52 | 10.61 | 6.41 | 6.66 | 6.10 | 12.08 | 12.89 | 20.33 | 11.11 |
| **Zimbabwe** | 1.33 | 2.19 | 1.41 | 2.26 | 1.97 | 2.61 | 1.94 | 7.22 | 3.95 | 6.31 | 8.74 | 7.61 | 17.22 | 13.71 | 12.74 |

*2. Health*

|  | **2000** | **2001** | **2002** | **2003** | **2004** | **2005** | **2006** | **2007** | **2008** | **2009** | **2010** | **2011** | **2012** | **2013** | **2014** |
| --- | --- | --- | --- | --- | --- | --- | --- | --- | --- | --- | --- | --- | --- | --- | --- |
| **Afghanistan** | 3.51 | 6.41 | 14.31 | 14.09 | 21.42 | 26.01 | 16.02 | 40.62 | 25.86 | 22.97 | 22.20 | 22.73 | 18.40 | 21.26 | 20.31 |
| **Angola** | 5.12 | 8.05 | 5.57 | 5.21 | 5.00 | 9.06 | 5.05 | 6.26 | 7.60 | 6.07 | 7.14 | 4.87 | 6.36 | 7.09 | 5.22 |
| **Azerbaijan** | 1.07 | 0.97 | 1.74 | 0.96 | 1.17 | 3.47 | 3.59 | 3.47 | 2.76 | 3.75 | 4.26 | 6.95 | 3.24 | 4.47 | 4.39 |
| **Bangladesh** | 2.61 | 2.74 | 2.13 | 2.52 | 2.16 | 2.26 | 2.91 | 1.85 | 2.35 | 3.01 | 2.99 | 3.08 | 3.41 | 4.83 | 3.98 |
| **Benin** | 8.44 | 9.26 | 7.27 | 7.99 | 10.06 | 10.55 | 9.51 | 9.41 | 11.49 | 16.11 | 16.76 | 17.42 | 11.66 | 13.06 | 15.46 |
| **Bolivia** | 14.12 | 19.21 | 31.24 | 57.52 | 21.95 | 17.70 | 25.43 | 21.46 | 14.70 | 13.21 | 10.27 | 10.80 | 8.73 | 9.47 | 7.30 |
| **Botswana** | 7.43 | 7.55 | 12.67 | 15.30 | 28.29 | 17.96 | 24.08 | 37.23 | 168.29 | 172.86 | 57.76 | 58.15 | 44.47 | 50.68 | 37.32 |
| **Brazil** | 0.15 | 0.27 | 0.31 | 0.35 | 0.32 | 0.24 | 0.23 | 0.17 | 0.20 | 0.26 | 0.29 | 0.24 | 0.13 | 0.14 | 0.10 |
| **Burkina Faso** | 5.16 | 5.79 | 6.61 | 8.60 | 8.84 | 10.57 | 10.60 | 8.52 | 10.29 | 13.74 | 15.02 | 8.77 | 12.42 | 12.06 | 11.82 |
| **Burundi** | 5.14 | 3.81 | 4.22 | 6.48 | 6.29 | 6.94 | 8.59 | 8.03 | 10.19 | 16.55 | 14.24 | 14.73 | 12.57 | 14.65 | 14.55 |
| **Cambodia** | 8.48 | 7.98 | 6.60 | 9.77 | 8.78 | 12.22 | 11.46 | 12.90 | 13.10 | 17.63 | 21.31 | 18.63 | 15.26 | 16.33 | 20.21 |
| **Cameroon** | 1.60 | 2.77 | 2.92 | 3.36 | 4.61 | 4.78 | 5.78 | 5.88 | 4.45 | 6.25 | 4.27 | 9.17 | 6.24 | 9.51 | 7.63 |
| **Central African Republic** | 6.71 | 3.92 | 6.88 | 4.86 | 8.39 | 7.08 | 7.43 | 4.61 | 10.83 | 5.69 | 8.98 | 8.66 | 8.57 | 10.34 | 16.87 |
| **Chad** | 6.04 | 5.90 | 7.71 | 7.07 | 7.20 | 8.06 | 4.61 | 3.88 | 4.65 | 4.55 | 8.14 | 6.42 | 4.91 | 10.61 | 7.94 |
| **China** | 0.23 | 0.17 | 0.19 | 0.19 | 0.23 | 0.20 | 0.29 | 0.38 | 0.27 | 0.35 | 0.29 | 0.24 | 0.27 | 0.14 | 0.12 |
| **Comoros** | 8.76 | 8.58 | 17.80 | 19.25 | 12.61 | 7.87 | 6.65 | 6.48 | 7.73 | 9.78 | 22.80 | 17.19 | 41.21 | 23.49 | 17.40 |
| **Congo** | 4.03 | 7.18 | 2.97 | 5.92 | 8.49 | 6.26 | 5.67 | 6.36 | 8.79 | 6.12 | 17.08 | 14.89 | 13.56 | 9.65 | 4.73 |
| **Cote d'Ivoire** | 1.98 | 2.24 | 4.82 | 4.19 | 4.10 | 4.26 | 5.36 | 5.71 | 9.60 | 8.68 | 12.49 | 8.20 | 9.89 | 10.74 | 13.36 |
| **DPR of Korea** | 0.26 | 0.23 | 0.48 | 0.42 | 0.67 | 0.89 | 0.38 | 0.43 | 0.32 | 1.10 | 1.79 | 1.67 | 1.50 | 1.55 | 2.09 |
| **DR Congo** | 1.57 | 2.30 | 3.00 | 3.65 | 3.26 | 4.52 | 4.25 | 4.58 | 8.02 | 8.29 | 9.62 | 10.42 | 11.57 | 11.84 | 11.80 |
| **Djibouti** | 24.38 | 9.17 | 12.42 | 7.88 | 19.65 | 31.00 | 36.10 | 38.99 | 28.44 | 29.93 | 17.88 | 18.73 | 28.35 | 21.39 | 17.81 |
| **Egypt** | 2.99 | 3.06 | 2.06 | 1.77 | 2.84 | 1.92 | 2.66 | 1.84 | 2.71 | 1.12 | 1.19 | 0.94 | 1.00 | 1.07 | 0.75 |
| **Equatorial Guinea** | 24.73 | 16.83 | 13.40 | 13.54 | 26.12 | 25.25 | 29.50 | 21.62 | 33.00 | 28.97 | 30.07 | 19.05 | 8.30 | 2.91 | 4.89 |
| **Eritrea** | 6.18 | 8.67 | 14.42 | 16.63 | 16.52 | 15.71 | 6.97 | 10.22 | 10.38 | 9.04 | 17.78 | 7.13 | 9.55 | 10.21 | 10.27 |
| **Ethiopia** | 2.38 | 3.77 | 2.88 | 4.58 | 4.19 | 4.97 | 9.78 | 10.31 | 8.69 | 10.04 | 12.40 | 13.50 | 11.71 | 16.16 | 12.51 |
| **Gabon** | 9.84 | 11.00 | 10.79 | 6.65 | 8.76 | 10.51 | 13.13 | 8.73 | 6.98 | 11.99 | 6.35 | 8.40 | 6.71 | 9.27 | 8.78 |
| **Gambia** | 9.76 | 9.62 | 13.51 | 13.34 | 19.27 | 24.06 | 14.27 | 33.77 | 11.89 | 16.58 | 25.53 | 27.23 | 26.98 | 34.02 | 24.06 |
| **Ghana** | 5.92 | 7.98 | 7.09 | 10.22 | 14.30 | 14.62 | 11.74 | 11.33 | 12.18 | 15.46 | 15.16 | 13.40 | 16.97 | 16.21 | 12.75 |
| **Guatemala** | 5.14 | 7.11 | 8.35 | 6.93 | 5.38 | 3.75 | 4.80 | 5.56 | 7.29 | 6.32 | 5.34 | 7.41 | 5.61 | 6.12 | 6.10 |
| **Guinea** | 5.75 | 11.93 | 6.04 | 4.90 | 5.03 | 5.68 | 5.36 | 4.22 | 4.35 | 4.70 | 6.11 | 5.14 | 8.36 | 5.43 | 21.11 |
| **Guinea-Bissau** | 7.70 | 10.63 | 11.51 | 10.68 | 11.80 | 14.94 | 14.67 | 20.97 | 13.31 | 17.37 | 28.36 | 24.09 | 16.44 | 32.17 | 22.28 |
| **Haiti** | 11.00 | 10.63 | 5.55 | 9.07 | 14.72 | 11.88 | 18.47 | 22.94 | 23.12 | 26.12 | 27.45 | 39.24 | 31.27 | 31.50 | 29.67 |
| **India** | 0.85 | 0.87 | 1.10 | 0.68 | 0.81 | 0.97 | 0.63 | 0.94 | 0.85 | 1.12 | 1.24 | 1.46 | 0.99 | 1.17 | 1.04 |
| **Indonesia** | 1.00 | 0.98 | 0.72 | 1.04 | 1.09 | 1.37 | 1.96 | 1.30 | 1.48 | 1.57 | 1.45 | 1.37 | 1.66 | 1.48 | 1.00 |
| **Iraq** | 0.35 | 0.22 | 0.30 | 3.39 | 4.62 | 23.79 | 17.18 | 11.51 | 3.64 | 3.87 | 3.77 | 1.57 | 1.93 | 1.61 | 2.20 |
| **Kenya** | 5.13 | 6.05 | 7.36 | 8.50 | 9.39 | 9.63 | 13.11 | 13.86 | 16.74 | 22.19 | 25.27 | 27.31 | 28.56 | 29.96 | 26.20 |
| **Kyrgyzstan** | 5.34 | 2.83 | 5.83 | 7.71 | 6.25 | 11.38 | 14.16 | 16.88 | 20.85 | 11.61 | 13.02 | 19.72 | 14.94 | 16.28 | 23.83 |
| **Lao** | 7.72 | 6.73 | 11.43 | 10.78 | 7.44 | 10.05 | 13.97 | 10.27 | 12.82 | 10.83 | 13.57 | 14.84 | 13.76 | 18.02 | 27.06 |
| **Lesotho** | 8.74 | 8.64 | 8.55 | 10.72 | 13.52 | 14.46 | 13.27 | 21.58 | 28.89 | 30.85 | 51.98 | 74.19 | 66.74 | 82.98 | 34.67 |
| **Liberia** | 3.99 | 4.65 | 3.49 | 5.73 | 8.18 | 12.42 | 10.40 | 10.69 | 22.23 | 29.92 | 34.23 | 33.83 | 37.79 | 34.06 | 53.93 |
| **Madagascar** | 3.82 | 4.07 | 4.22 | 6.35 | 6.70 | 7.19 | 7.60 | 7.59 | 6.93 | 5.82 | 10.04 | 6.54 | 6.18 | 8.66 | 7.71 |
| **Malawi** | 19.82 | 11.90 | 14.68 | 16.74 | 16.56 | 16.09 | 21.36 | 26.26 | 27.07 | 30.06 | 23.78 | 32.99 | 34.99 | 35.09 | 30.91 |
| **Mali** | 7.85 | 7.39 | 4.73 | 7.71 | 7.40 | 10.53 | 11.26 | 12.36 | 12.70 | 15.26 | 16.64 | 18.74 | 16.03 | 16.60 | 17.05 |
| **Mauritania** | 11.24 | 9.38 | 12.46 | 11.21 | 9.71 | 6.01 | 15.71 | 11.02 | 9.50 | 8.07 | 7.31 | 7.98 | 5.27 | 8.83 | 8.72 |
| **Mexico** | 0.30 | 0.25 | 0.90 | 0.80 | 0.60 | 0.56 | 0.92 | 0.46 | 0.29 | 0.21 | 0.21 | 0.25 | 0.26 | 0.22 | 0.08 |
| **Morocco** | 2.01 | 0.94 | 1.17 | 2.90 | 4.19 | 3.61 | 5.38 | 4.81 | 5.41 | 3.45 | 3.23 | 2.61 | 2.15 | 3.98 | 4.96 |
| **Mozambique** | 15.49 | 13.81 | 13.85 | 14.04 | 17.57 | 15.76 | 22.48 | 21.88 | 23.78 | 26.67 | 29.34 | 28.44 | 27.75 | 29.50 | 34.94 |
| **Myanmar** | 0.36 | 0.62 | 0.86 | 1.18 | 1.16 | 1.47 | 1.01 | 1.48 | 1.57 | 2.15 | 3.28 | 2.52 | 4.58 | 6.05 | 7.43 |
| **Nepal** | 3.33 | 4.20 | 3.55 | 4.22 | 3.29 | 4.00 | 4.84 | 4.77 | 5.79 | 6.65 | 8.20 | 6.59 | 6.45 | 7.97 | 9.25 |
| **Niger** | 4.21 | 4.92 | 5.32 | 6.18 | 5.06 | 5.52 | 13.19 | 7.68 | 9.51 | 9.44 | 9.30 | 7.96 | 5.21 | 7.65 | 10.04 |
| **Nigeria** | 1.58 | 1.57 | 2.15 | 2.01 | 3.54 | 3.48 | 4.22 | 5.51 | 6.33 | 9.85 | 7.06 | 7.94 | 8.68 | 11.21 | 9.57 |
| **Pakistan** | 0.80 | 1.61 | 2.48 | 1.54 | 1.69 | 1.99 | 2.82 | 3.51 | 3.46 | 3.34 | 3.98 | 2.72 | 3.59 | 4.50 | 2.88 |
| **Papua New Guinea** | 19.90 | 19.07 | 25.83 | 22.08 | 22.71 | 28.19 | 20.82 | 15.46 | 17.82 | 24.47 | 24.24 | 33.04 | 31.16 | 31.71 | 29.58 |
| **Peru** | 2.09 | 2.35 | 8.48 | 14.30 | 5.14 | 6.65 | 9.63 | 4.09 | 5.22 | 7.86 | 3.98 | 2.42 | 3.21 | 2.64 | 2.18 |
| **Philippines** | 1.03 | 1.07 | 0.91 | 1.33 | 1.21 | 1.16 | 1.31 | 1.65 | 1.18 | 1.52 | 2.14 | 1.76 | 1.27 | 1.40 | 1.68 |
| **Rwanda** | 10.63 | 9.91 | 10.54 | 9.94 | 16.18 | 18.06 | 27.29 | 26.18 | 34.18 | 39.62 | 43.86 | 45.19 | 40.25 | 34.55 | 40.13 |
| **Sao Tome and Principe** | 33.14 | 99.82 | 88.86 | 54.35 | 52.13 | 56.19 | 47.86 | 41.91 | 53.54 | 34.71 | 43.42 | 81.08 | 51.07 | 85.10 | 72.87 |
| **Senegal** | 6.70 | 12.69 | 10.14 | 16.62 | 17.07 | 15.52 | 25.10 | 11.47 | 11.60 | 15.68 | 12.96 | 15.38 | 15.70 | 14.50 | 17.83 |
| **Sierra Leone** | 8.16 | 6.52 | 8.76 | 10.27 | 9.92 | 13.35 | 11.17 | 13.07 | 14.18 | 18.67 | 22.57 | 17.77 | 17.30 | 20.88 | 38.02 |
| **Solomon Islands** | 9.38 | 35.35 | 44.65 | 57.29 | 57.94 | 68.33 | 52.02 | 45.16 | 58.05 | 74.50 | 74.64 | 94.14 | 48.22 | 88.59 | 27.18 |
| **Somalia** | 3.46 | 10.45 | 4.81 | 2.59 | 3.97 | 5.50 | 4.55 | 5.37 | 4.47 | 5.73 | 7.18 | 9.00 | 14.55 | 15.12 | 16.93 |
| **South Africa** | 4.13 | 4.21 | 2.16 | 3.88 | 5.04 | 5.95 | 6.29 | 10.88 | 13.96 | 19.41 | 17.83 | 19.12 | 18.31 | 18.00 | 17.33 |
| **Sudan** | 1.60 | 1.89 | 2.29 | 1.97 | 2.90 | 5.67 | 4.62 | 5.11 | 5.84 | 5.44 | 8.75 | 6.42 | 6.24 | 6.00 | 4.57 |
| **Swaziland** | 5.87 | 5.48 | 8.24 | 20.46 | 14.64 | 35.54 | 20.75 | 28.52 | 30.39 | 53.68 | 83.27 | 97.74 | 71.02 | 82.78 | 55.28 |
| **Tajikistan** | 3.20 | 4.44 | 3.01 | 3.54 | 4.83 | 6.62 | 6.49 | 7.47 | 13.23 | 6.54 | 10.89 | 8.48 | 9.56 | 9.32 | 10.27 |
| **Tanzania** | 8.99 | 7.74 | 7.04 | 7.16 | 9.71 | 11.13 | 13.92 | 15.20 | 18.65 | 19.53 | 24.18 | 21.92 | 22.22 | 26.32 | 24.55 |
| **Togo** | 5.55 | 2.38 | 2.48 | 4.05 | 5.14 | 5.36 | 4.30 | 6.80 | 7.66 | 9.01 | 8.43 | 11.25 | 4.81 | 10.93 | 8.56 |
| **Turkmenistan** | 7.28 | 11.93 | 4.11 | 2.28 | 6.25 | 4.71 | 6.62 | 4.07 | 4.18 | 1.62 | 2.46 | 2.86 | 1.35 | 2.54 | 2.60 |
| **Uganda** | 13.33 | 11.45 | 9.90 | 15.31 | 18.08 | 17.22 | 18.61 | 18.82 | 15.55 | 21.04 | 19.21 | 20.04 | 26.03 | 22.55 | 23.69 |
| **Uzbekistan** | 1.22 | 1.61 | 2.47 | 1.88 | 1.75 | 1.42 | 1.79 | 3.16 | 1.92 | 2.61 | 2.30 | 2.34 | 3.32 | 1.98 | 2.73 |
| **Viet Nam** | 2.10 | 1.78 | 1.79 | 2.27 | 1.92 | 2.44 | 2.81 | 2.89 | 3.32 | 4.49 | 4.83 | 5.30 | 4.71 | 5.13 | 4.08 |
| **Yemen** | 6.69 | 4.31 | 4.97 | 5.40 | 2.14 | 3.92 | 3.12 | 3.32 | 3.39 | 3.07 | 3.97 | 4.34 | 5.11 | 7.44 | 8.37 |
| **Zambia** | 11.90 | 11.85 | 19.52 | 28.12 | 29.44 | 31.45 | 31.40 | 37.27 | 42.71 | 39.62 | 31.50 | 44.92 | 43.44 | 52.93 | 39.10 |
| **Zimbabwe** | 5.46 | 5.74 | 6.50 | 8.59 | 9.21 | 14.32 | 12.04 | 17.27 | 11.72 | 18.22 | 24.46 | 19.87 | 41.44 | 30.51 | 30.32 |

1. *Food and humanitarian assistance*

|  | **2000** | **2001** | **2002** | **2003** | **2004** | **2005** | **2006** | **2007** | **2008** | **2009** | **2010** | **2011** | **2012** | **2013** | **2014** |
| --- | --- | --- | --- | --- | --- | --- | --- | --- | --- | --- | --- | --- | --- | --- | --- |
| **Afghanistan** | 5.94 | 18.8 | 69.2 | 39.4 | 30.6 | 31.6 | 27.9 | 39.7 | 63.5 | 85.3 | 42.2 | 66.8 | 27.4 | 25.4 | 24.5 |
| **Angola** | 16.32 | 16.5 | 31.5 | 31.7 | 23.3 | 13.1 | 8.31 | 4.58 | 2.21 | 1.56 | 0.95 | 1.22 | 1.09 | 0.84 | 1.69 |
| **Azerbaijan** | 3.68 | 4.3 | 5.49 | 6.05 | 5.68 | 8.74 | 4.11 | 2.62 | 2.94 | 1.94 | 2.03 | 9.21 | 1.55 | 2.21 | 3.16 |
| **Bangladesh** | 1.09 | 1.15 | 1.22 | 0.8 | 1.37 | 1.56 | 1.28 | 1.55 | 4.23 | 3.31 | 1.86 | 1.61 | 1.6 | 1.14 | 1.44 |
| **Benin** | 1.82 | 1.47 | 2.28 | 1.85 | 1.75 | 2.22 | 1.49 | 0.68 | 0.61 | 2.14 | 2.4 | 1.95 | 2.51 | 1.55 | 1.87 |
| **Bolivia** | 6.17 | 5.37 | 6.03 | 12.4 | 7.49 | 5.97 | 7.48 | 8.37 | 6.67 | 3.95 | 2.2 | 1.77 | 0.88 | 1.26 | 1.06 |
| **Botswana** | 1.10 | 2.13 | 1.81 | 1.39 | 1.74 | 0.63 | 0.33 | 0.8 | 0.8 | 0.89 | 0.7 | 1.72 | 0.12 | 0.26 | 0.1 |
| **Brazil** | 0.04 | 0.03 | 0.04 | 0.05 | 0.03 | 0.04 | 0.04 | 0.03 | 0.06 | 0.03 | 0.03 | 0.05 | 0.02 | 0.02 | 0.02 |
| **Burkina Faso** | 2.87 | 3.38 | 4.28 | 2.83 | 1.84 | 2.74 | 3.22 | 4.21 | 4.17 | 5.63 | 5.52 | 4.28 | 8.54 | 7.62 | 6.86 |
| **Burundi** | 6.94 | 13.5 | 17.9 | 23.9 | 26.7 | 28 | 26.6 | 18.9 | 16 | 17 | 12.7 | 8.39 | 6.76 | 6.04 | 5.16 |
| **Cambodia** | 10.70 | 6.44 | 7.61 | 4.24 | 2.74 | 5.66 | 4.07 | 4.68 | 4.19 | 4.54 | 3.73 | 5.25 | 4.2 | 2.73 | 3.46 |
| **Cameroon** | 0.28 | 0.46 | 0.42 | 1.09 | 0.72 | 1.19 | 0.68 | 0.56 | 1.35 | 2.03 | 1.23 | 1.24 | 1.75 | 1.51 | 6.04 |
| **Central African Republic** | 2.63 | 2.72 | 3.25 | 2.79 | 3.78 | 3.35 | 4.81 | 19.7 | 22 | 18.5 | 16.2 | 17.9 | 17.9 | 28.7 | 86.7 |
| **Chad** | 2.22 | 2.77 | 1.79 | 3.09 | 16 | 19.8 | 15.5 | 25.6 | 28.6 | 38 | 31.2 | 24.6 | 30 | 20.7 | 18.1 |
| **China** | 0.07 | 0.06 | 0.07 | 0.09 | 0.02 | 0.06 | 0 | 0.01 | 0.45 | 0.02 | 0.03 | 0.03 | 0.01 | 0.08 | 0.01 |
| **Comoros** | 0.98 | 0.93 | 0.48 | 0.32 | 0.25 | 6.55 | 2.42 | 0.86 | 3.04 | 9.89 | 1.87 | 2.96 | 13.7 | 0.52 | 9.8 |
| **Congo** | 5.01 | 11.3 | 15.4 | 13.3 | 8.38 | 12.8 | 6.49 | 5.66 | 4.1 | 2.36 | 11.7 | 2.94 | 3.59 | 4.03 | 1.33 |
| **Cote d'Ivoire** | 0.91 | 1.11 | 1.47 | 4.47 | 5.41 | 5.05 | 5.13 | 4.05 | 7.05 | 3.13 | 2.44 | 13.2 | 6.65 | 3.8 | 4.57 |
| **Democratic People's Republic of Korea** | 5.42 | 9.19 | 13.6 | 6.97 | 8 | 3.6 | 2.18 | 2.99 | 6.54 | 1.89 | 1.58 | 32.1 | 3.23 | 11 | 28.8 |
| **Democratic Republic of the Congo** | 3.23 | 4.11 | 8.25 | 7.33 | 7.24 | 8.3 | 10.7 | 9.85 | 12.2 | 12.9 | 10.6 | 8.68 | 9.37 | 8.63 | 9.41 |
| **Djibouti** | 12.85 | 13.7 | 16.8 | 19.1 | 12.2 | 16.8 | 19.6 | 25.8 | 28 | 33.1 | 27.3 | 50.7 | 29.5 | 32.2 | 59.1 |
| **Egypt** | 0.31 | 0.11 | 0.24 | 0.26 | 0.16 | 0.12 | 0.15 | 0.08 | 0.1 | 0.16 | 0.17 | 0.51 | 3.06 | 3.54 | 0.34 |
| **Equatorial Guinea** | 0.20 | 0.09 | 0.15 | 0.13 | 0.16 | 0.32 | 0.17 | 0.13 | 3.37 | 1.68 | 0.02 | 0.1 | 0.01 | 0.03 | 0.34 |
| **Eritrea** | 30.65 | 31.2 | 35.3 | 68.3 | 53.3 | 65 | 17.2 | 15.1 | 13 | 14.9 | 12.3 | 5.96 | 3.04 | 2.65 | 3.46 |
| **Ethiopia** | 6.21 | 7.15 | 8.11 | 17.4 | 10.5 | 13.9 | 9.44 | 8.56 | 18.3 | 14.1 | 13.9 | 11.4 | 9.5 | 9.16 | 8.49 |
| **Gabon** | 3.00 | 4.69 | 3.44 | 3.77 | 3.65 | 1.27 | 0.12 | 0.61 | 0.59 | 0.76 | 0.67 | 0.25 | 0.06 | 0.34 | 0.13 |
| **Gambia** | 5.02 | 3.06 | 2.63 | 7.31 | 3.06 | 3.97 | 7.91 | 5.01 | 11.6 | 9.36 | 7.76 | 7.64 | 3.86 | 7.31 | 2.48 |
| **Ghana** | 1.51 | 1.39 | 2.17 | 2.25 | 1.53 | 1.96 | 1.58 | 2.64 | 1.5 | 1.61 | 0.45 | 0.72 | 0.6 | 0.24 | 0.5 |
| **Guatemala** | 4.95 | 4.42 | 6.13 | 5.31 | 3.47 | 7.77 | 8.88 | 4.5 | 3.8 | 4.56 | 5.98 | 5.56 | 3.58 | 2.18 | 2.39 |
| **Guinea** | 6.53 | 11.5 | 11.3 | 8.63 | 6.77 | 5.72 | 3.93 | 4.51 | 2.85 | 3.47 | 1.77 | 2.66 | 1.59 | 1.83 | 7.24 |
| **Guinea-Bissau** | 7.14 | 7.15 | 6.7 | 6.42 | 5.5 | 17.9 | 8.76 | 10.8 | 10.4 | 8.52 | 4.18 | 5.09 | 2.93 | 3.78 | 6.03 |
| **Haiti** | 7.77 | 7.09 | 5.38 | 10.5 | 18.6 | 25.3 | 17.8 | 14.1 | 34.1 | 28.8 | 203 | 68.1 | 39.3 | 31.4 | 20.2 |
| **India** | 0.24 | 0.34 | 0.27 | 0.15 | 0.19 | 0.18 | 0.14 | 0.08 | 0.09 | 0.06 | 0.04 | 0.04 | 0.05 | 0.06 | 0.11 |
| **Indonesia** | 0.86 | 0.63 | 0.74 | 0.7 | 0.5 | 4.8 | 2.85 | 1.47 | 1.17 | 1.41 | 0.69 | 0.82 | 0.25 | 0.42 | 0.23 |
| **Iraq** | 3.30 | 3.34 | 4.15 | 59.3 | 59.6 | 43.7 | 23 | 29.7 | 46.4 | 27.1 | 14.5 | 12.1 | 4.27 | 6.27 | 21.7 |
| **Kenya** | 3.94 | 6.08 | 6.44 | 4.55 | 4.46 | 3.73 | 9.84 | 7.73 | 10.4 | 13.7 | 9.87 | 14.7 | 12.9 | 11.6 | 11.3 |
| **Kyrgyzstan** | 5.14 | 3.68 | 4.4 | 3.45 | 6.01 | 10.9 | 8.41 | 4.56 | 4.99 | 5.02 | 20 | 90.1 | 23.6 | 108 | 359 |
| **Lao People's Democratic Republic** | 4.94 | 7.15 | 8.21 | 5.29 | 2.71 | 3.84 | 4.62 | 4.48 | 4.64 | 5.13 | 5.14 | 6.12 | 5.59 | 6.28 | 7.34 |
| **Lesotho** | 1.07 | 0.67 | 8.23 | 2.75 | 4.86 | 4.75 | 2.56 | 18.7 | 5.99 | 4.26 | 6.4 | 3.47 | 6.45 | 8.69 | 4.45 |
| **Liberia** | 31.77 | 16.2 | 27.6 | 43 | 92.1 | 54.1 | 65.4 | 57.9 | 62.7 | 25.4 | 18.9 | 37.5 | 30.3 | 17.4 | 41.9 |
| **Madagascar** | 1.88 | 2.32 | 1.74 | 1.89 | 1.76 | 3.39 | 3.04 | 4.11 | 3.06 | 3.05 | 3.75 | 2.43 | 2.79 | 1.77 | 2.03 |
| **Malawi** | 3.16 | 2.7 | 6.03 | 9.05 | 3.62 | 7.83 | 8.32 | 5.65 | 5.31 | 6.42 | 5.48 | 5.89 | 7.5 | 9.31 | 5.54 |
| **Mali** | 0.65 | 2.45 | 2.51 | 2.81 | 0.58 | 4.15 | 3.15 | 2.81 | 2.03 | 3.81 | 4 | 2.83 | 22.9 | 20.5 | 20.6 |
| **Mauritania** | 6.65 | 9.47 | 10.4 | 18.4 | 9.88 | 23.9 | 10.2 | 13.6 | 17.2 | 11.7 | 7.54 | 8.63 | 30.1 | 24 | 20.6 |
| **Mexico** | 0.10 | 0.09 | 0.05 | 0.05 | 0.03 | 0.04 | 0.02 | 0.06 | 0.05 | 0.08 | 0.03 | 0.02 | 0.02 | 0.02 | 0.02 |
| **Morocco** | 0.19 | 0.16 | 0.14 | 0.13 | 1.1 | 0.47 | 0.51 | 0.75 | 0.4 | 0.13 | 0.27 | 0.38 | 0.79 | 0.41 | 0.84 |
| **Mozambique** | 17.09 | 10 | 5.57 | 5.6 | 3.46 | 3.27 | 3.82 | 6.61 | 5.37 | 5.94 | 2.91 | 3.28 | 4.01 | 4.29 | 1.92 |
| **Myanmar** | 0.63 | 0.62 | 0.91 | 0.56 | 0.8 | 1.14 | 1.01 | 1.37 | 8.89 | 4.27 | 3.15 | 2.56 | 3.06 | 5.12 | 4.87 |
| **Nepal** | 2.62 | 2.15 | 2.93 | 2.52 | 2 | 2.52 | 3.35 | 3.96 | 4.19 | 4.22 | 1.7 | 3.9 | 2.4 | 1.53 | 1.03 |
| **Niger** | 2.35 | 3.72 | 3.51 | 2.2 | 2.37 | 11 | 9.32 | 7.94 | 7.98 | 7.17 | 20.9 | 14.1 | 25.8 | 15.1 | 16.2 |
| **Nigeria** | 0.04 | 0.04 | 0.12 | 0.09 | 0.09 | 0.18 | 0.03 | 0.06 | 0.05 | 0.07 | 0.11 | 0.14 | 0.16 | 0.18 | 0.35 |
| **Pakistan** | 0.79 | 1.11 | 1.35 | 1 | 0.87 | 4.76 | 8.05 | 3.1 | 1.55 | 4.73 | 10.5 | 13 | 4.37 | 2.88 | 2.46 |
| **Papua New Guinea** | 3.43 | 0.48 | 1.71 | 1.95 | 0.6 | 1.42 | 0.37 | 0.82 | 1.41 | 2.18 | 2.59 | 1.53 | 0.79 | 0.95 | 0.84 |
| **Peru** | 7.09 | 10.3 | 3.61 | 2.72 | 2.16 | 0.88 | 1.66 | 5.21 | 2.15 | 1.03 | 0.69 | 1.08 | 0.43 | 0.84 | 0.64 |
| **Philippines** | 2.39 | 2.14 | 0.91 | 0.45 | 0.25 | 0.63 | 0.78 | 0.39 | 0.59 | 1.55 | 1.57 | 1.33 | 1.63 | 4.84 | 7.65 |
| **Rwanda** | 14.58 | 7.35 | 8.74 | 9.88 | 9.24 | 8.17 | 5.51 | 5.82 | 3.78 | 4.54 | 2.02 | 3.52 | 3.93 | 4.31 | 3.09 |
| **Sao Tome and Principe** | 11.42 | 10.4 | 15.6 | 11.8 | 10.5 | 11.6 | 1.25 | 14.1 | 50.4 | 4.89 | 24.3 | 21 | 19.3 | 19.1 | 26.7 |
| **Senegal** | 3.40 | 3.33 | 2.76 | 2.1 | 2.39 | 1.98 | 1.94 | 3.13 | 4.08 | 3.37 | 4.24 | 2.83 | 5.82 | 5.65 | 3.03 |
| **Sierra Leone** | 13.49 | 27.9 | 75.4 | 41.4 | 23.7 | 15.1 | 13.9 | 12.8 | 8.13 | 7.44 | 4.74 | 7.3 | 7.19 | 5.75 | 81.7 |
| **Solomon Islands** | 8.07 | 4.79 | 33.6 | 33.5 | 36.2 | 6.89 | 5.51 | 19.3 | 12.2 | 8.92 | 18.1 | 20.6 | 2.46 | 20.7 | 24.1 |
| **Somalia** | 14.47 | 17.4 | 22.2 | 14 | 17.9 | 25.6 | 51.6 | 44.5 | 83.4 | 68.9 | 39.2 | 94.8 | 73.1 | 67.4 | 74.4 |
| **South Africa** | 0.26 | 0.29 | 0.3 | 0.48 | 0.11 | 0.13 | 0.19 | 0.12 | 0.29 | 0.25 | 0.23 | 0.45 | 0.42 | 0.21 | 0.14 |
| **Sudan** | 3.73 | 5.06 | 13.7 | 17.7 | 41.4 | 57.2 | 60.7 | 56.4 | 54.8 | 50.5 | 36.2 | 30 | 24.6 | 31 | 17.2 |
| **Swaziland** | 0.60 | 0.55 | 1.91 | 1.82 | 0.66 | 1.91 | 0.48 | 13.1 | 5.95 | 3.36 | 5.51 | 3.79 | 4.36 | 3.27 | 4.79 |
| **Tajikistan** | 10.35 | 17.8 | 16.7 | 12.2 | 7.24 | 10.2 | 7.98 | 4.35 | 6.76 | 19.4 | 7.33 | 28.7 | 6.12 | 15.9 | 25.5 |
| **Tanzania** | 2.85 | 3.61 | 3.53 | 3.54 | 1.79 | 1.9 | 2.41 | 3.82 | 2.28 | 1.95 | 1.47 | 1.76 | 1.49 | 1.52 | 1.38 |
| **Togo** | 0.46 | 0.59 | 0.24 | 0.72 | 0.06 | 1.12 | 0.31 | 1.03 | 1.25 | 3.01 | 0.69 | 1.67 | 2.5 | 1.37 | 1.12 |
| **Turkmenistan** | 3.71 | 6.58 | 3.36 | 0.81 | 4.92 | 2.26 | 7.35 | 0.66 | 1.07 | 0.78 | 1.47 | 1.78 | 1.88 | 1.72 | 1.47 |
| **Uganda** | 3.56 | 4.68 | 5.26 | 9.52 | 10.9 | 10.2 | 12.1 | 12.4 | 11.9 | 8.58 | 4.71 | 3.57 | 3.07 | 3.29 | 3 |
| **Uzbekistan** | 3.36 | 2.85 | 1.38 | 1.33 | 0.49 | 0.32 | 0.19 | 0.12 | 0.92 | 0.41 | 0.56 | 0.52 | 0.19 | 0.26 | 0.53 |
| **Viet Nam** | 0.62 | 0.85 | 0.61 | 1.07 | 0.43 | 0.37 | 0.55 | 0.23 | 0.39 | 0.93 | 0.49 | 0.74 | 0.82 | 1.02 | 0.33 |
| **Yemen** | 3.77 | 3.1 | 3.4 | 3.59 | 5.5 | 2.47 | 2.95 | 1.47 | 31.2 | 9.66 | 6.58 | 12.4 | 13.9 | 23.5 | 18.1 |
| **Zambia** | 7.16 | 4.97 | 9.11 | 6.6 | 5.45 | 5.45 | 4.23 | 4.23 | 4.84 | 4.91 | 2.74 | 3.24 | 1.3 | 3.63 | 0.79 |
| **Zimbabwe** | 3.00 | 2.12 | 9.67 | 8.93 | 5.43 | 20 | 10.5 | 19.9 | 35.5 | 39.1 | 21.5 | 14.9 | 13.4 | 10.4 | 8.59 |

1. *Water and Sanitation*

|  | **2000** | **2001** | **2002** | **2003** | **2004** | **2005** | **2006** | **2007** | **2008** | **2009** | **2010** | **2011** | **2012** | **2013** | **2014** |
| --- | --- | --- | --- | --- | --- | --- | --- | --- | --- | --- | --- | --- | --- | --- | --- |
| **Afghanistan** | 0.19 | 0.30 | 1.28 | 1.44 | 2.18 | 3.46 | 1.78 | 2.51 | 2.43 | 2.88 | 2.48 | 5.53 | 3.25 | 2.73 | 2.27 |
| **Angola** | 0.36 | 0.73 | 1.53 | 0.68 | 0.78 | 1.19 | 0.93 | 2.56 | 1.25 | 0.86 | 0.47 | 0.62 | 1.31 | 1.18 | 1.19 |
| **Azerbaijan** | 2.18 | 2.04 | 2.57 | 1.51 | 1.22 | 1.67 | 2.62 | 2.82 | 1.93 | 2.65 | 1.89 | 2.45 | 3.29 | 5.57 | 5.76 |
| **Bangladesh** | 0.71 | 0.81 | 0.45 | 0.41 | 0.33 | 0.51 | 0.83 | 0.82 | 0.94 | 0.95 | 1.31 | 1.39 | 1.15 | 0.98 | 1.26 |
| **Benin** | 3.21 | 5.25 | 3.42 | 2.80 | 6.87 | 8.12 | 7.10 | 8.87 | 8.15 | 6.81 | 8.43 | 5.56 | 4.89 | 6.42 | 6.19 |
| **Bolivia** | 10.18 | 5.07 | 4.22 | 3.61 | 5.70 | 6.62 | 5.67 | 17.71 | 7.50 | 8.00 | 6.91 | 8.58 | 15.72 | 6.89 | 5.94 |
| **Botswana** | 9.37 | 6.24 | 1.84 | 0.13 | 0.23 | 0.06 | 0.10 | 0.76 | 1.20 | 0.01 | 0.48 | 0.29 | 0.43 | 0.14 | 0.16 |
| **Brazil** | 0.69 | 0.39 | 0.03 | 0.04 | 0.03 | 0.04 | 0.10 | 0.19 | 1.38 | 0.55 | 0.08 | 1.13 | 0.10 | 0.75 | 0.87 |
| **Burkina Faso** | 3.17 | 4.97 | 4.10 | 5.85 | 5.57 | 6.53 | 8.26 | 6.95 | 7.35 | 4.92 | 5.20 | 8.70 | 7.04 | 6.19 | 6.75 |
| **Burundi** | 1.54 | 0.58 | 0.79 | 0.69 | 0.71 | 0.89 | 1.09 | 1.84 | 2.03 | 3.23 | 3.85 | 3.55 | 2.53 | 2.30 | 1.51 |
| **Cambodia** | 1.34 | 2.83 | 3.87 | 3.69 | 3.18 | 2.03 | 1.21 | 1.35 | 2.15 | 1.37 | 2.11 | 3.40 | 3.99 | 4.06 | 3.11 |
| **Cameroon** | 1.22 | 0.60 | 1.08 | 0.61 | 0.36 | 0.38 | 0.28 | 0.38 | 1.02 | 0.43 | 1.11 | 2.04 | 1.07 | 0.88 | 4.15 |
| **Central African Republic** | 1.51 | 1.35 | 0.34 | 0.07 | 0.06 | 0.10 | 0.25 | 0.52 | 0.77 | 2.48 | 1.40 | 1.97 | 1.65 | 1.73 | 1.12 |
| **Chad** | 1.49 | 4.27 | 2.17 | 2.82 | 2.02 | 2.25 | 2.56 | 2.22 | 2.58 | 2.88 | 3.68 | 2.66 | 2.25 | 1.71 | 1.87 |
| **China** | 0.29 | 0.37 | 0.25 | 0.17 | 0.18 | 0.26 | 0.17 | 0.24 | 0.21 | 0.27 | 0.20 | 0.18 | 0.17 | 0.18 | 0.13 |
| **Comoros** | 0.95 | 3.57 | 4.12 | 2.72 | 1.37 | 2.31 | 3.14 | 2.97 | 2.22 | 2.31 | 1.78 | 3.91 | 8.51 | 5.28 | 10.71 |
| **Congo** | 0.04 | 0.06 | 0.00 | 2.53 | 0.96 | 1.09 | 0.62 | 0.41 | 0.72 | 0.35 | 2.44 | 1.09 | 0.85 | 2.39 | 1.89 |
| **Cote d'Ivoire** | 2.53 | 0.25 | 2.02 | 0.25 | 0.38 | 0.25 | 0.10 | 0.31 | 0.58 | 1.78 | 2.07 | 1.58 | 2.46 | 1.02 | 1.25 |
| **DPR Korea** | 0.02 | 0.03 | 0.05 | 0.10 | 0.59 | 0.35 | 0.13 | 0.06 | 0.14 | 0.05 | 0.36 | 0.31 | 0.33 | 0.14 | 0.18 |
| **DR Congo** | 0.01 | 0.06 | 0.12 | 0.11 | 0.21 | 0.48 | 0.49 | 0.56 | 0.80 | 1.18 | 1.76 | 1.11 | 1.55 | 2.37 | 1.49 |
| **Djibouti** | 2.33 | 4.03 | 9.32 | 10.61 | 5.22 | 3.86 | 1.37 | 3.12 | 5.36 | 9.95 | 12.27 | 15.24 | 28.10 | 36.00 | 34.22 |
| **Egypt** | 0.91 | 0.94 | 2.07 | 1.57 | 4.10 | 1.44 | 0.99 | 1.21 | 1.21 | 1.90 | 1.07 | 0.74 | 1.15 | 0.62 | 0.85 |
| **Equatorial Guinea** | 0.21 | 0.14 | 14.20 | 2.37 | 6.06 | 2.52 | 5.50 | 1.14 | 0.04 | 0.04 | 0.00 | 0.03 | 0.00 | 0.00 | 0.01 |
| **Eritrea** | 3.34 | 2.79 | 3.97 | 0.96 | 0.90 | 1.83 | 2.11 | 1.04 | 2.29 | 2.22 | 2.24 | 0.69 | 0.70 | 0.22 | 0.21 |
| **Ethiopia** | 0.54 | 0.86 | 0.59 | 0.73 | 0.65 | 0.69 | 1.49 | 1.12 | 1.32 | 1.64 | 1.59 | 1.79 | 2.27 | 2.10 | 2.43 |
| **Gabon** | 0.13 | 0.01 | 0.01 | 0.04 | 0.08 | 0.34 | 0.02 | 0.05 | 12.62 | 1.58 | 6.67 | 9.76 | 7.44 | 15.54 | 11.73 |
| **Gambia** | 3.83 | 1.40 | 1.36 | 0.80 | 1.84 | 2.23 | 6.23 | 18.16 | 7.08 | 3.58 | 3.70 | 4.38 | 2.32 | 0.29 | 0.34 |
| **Ghana** | 5.46 | 5.52 | 5.79 | 4.00 | 4.35 | 4.49 | 9.86 | 6.52 | 6.01 | 2.93 | 4.37 | 2.25 | 4.45 | 2.68 | 4.15 |
| **Guatemala** | 3.78 | 2.18 | 1.41 | 1.25 | 1.11 | 1.64 | 2.36 | 1.26 | 1.09 | 2.71 | 2.26 | 0.70 | 0.39 | 0.39 | 0.14 |
| **Guinea** | 4.30 | 3.16 | 2.01 | 3.59 | 3.26 | 3.30 | 2.48 | 1.82 | 1.66 | 1.26 | 1.36 | 1.14 | 3.49 | 0.81 | 0.82 |
| **Guinea-Bissau** | 1.14 | 1.66 | 4.77 | 5.72 | 3.38 | 1.90 | 1.76 | 3.12 | 2.33 | 1.87 | 1.12 | 3.93 | 3.54 | 2.56 | 1.77 |
| **Haiti** | 1.43 | 0.39 | 0.90 | 0.61 | 0.62 | 0.93 | 1.14 | 1.17 | 3.43 | 13.85 | 3.92 | 3.84 | 3.34 | 2.62 | 5.30 |
| **India** | 0.23 | 0.24 | 0.14 | 0.12 | 0.11 | 0.16 | 0.18 | 0.22 | 0.28 | 0.22 | 0.34 | 0.35 | 0.33 | 0.34 | 0.37 |
| **Indonesia** | 0.53 | 0.43 | 0.27 | 0.22 | 0.22 | 0.28 | 0.42 | 0.45 | 0.53 | 0.74 | 0.87 | 0.84 | 0.71 | 0.57 | 0.39 |
| **Iraq** | 0.07 | 0.07 | 0.09 | 1.26 | 3.58 | 40.14 | 26.62 | 18.01 | 4.44 | 4.40 | 5.83 | 2.11 | 2.35 | 1.90 | 4.25 |
| **Kenya** | 0.90 | 1.06 | 1.02 | 0.94 | 1.11 | 1.63 | 1.47 | 1.90 | 3.22 | 3.11 | 4.21 | 4.15 | 4.73 | 4.76 | 5.05 |
| **Kyrgyzstan** | 0.91 | 1.95 | 1.35 | 1.38 | 3.00 | 2.72 | 3.56 | 2.78 | 2.55 | 3.36 | 2.56 | 5.99 | 3.02 | 2.53 | 4.46 |
| **Lao** | 4.50 | 3.21 | 4.28 | 4.13 | 3.75 | 3.63 | 2.42 | 4.99 | 4.01 | 5.81 | 4.11 | 4.03 | 3.98 | 3.82 | 5.08 |
| **Lesotho** | 1.66 | 1.21 | 2.61 | 5.82 | 3.86 | 5.57 | 4.47 | 10.20 | 12.67 | 17.56 | 22.46 | 29.37 | 40.46 | 54.08 | 17.98 |
| **Liberia** | 0.10 | 0.16 | 0.08 | 0.00 | 0.12 | 0.33 | 0.15 | 0.20 | 1.32 | 2.54 | 2.23 | 1.13 | 2.49 | 5.49 | 3.80 |
| **Madagascar** | 1.33 | 0.93 | 0.83 | 1.33 | 1.51 | 1.70 | 2.24 | 0.89 | 1.03 | 0.86 | 0.62 | 0.99 | 0.84 | 1.29 | 1.12 |
| **Malawi** | 2.92 | 2.84 | 3.77 | 3.35 | 1.86 | 1.92 | 9.24 | 1.58 | 1.29 | 1.51 | 1.80 | 2.46 | 4.75 | 6.58 | 6.11 |
| **Mali** | 3.73 | 3.65 | 2.48 | 1.94 | 2.38 | 3.41 | 5.57 | 3.71 | 4.01 | 5.58 | 3.92 | 3.76 | 2.18 | 3.42 | 5.59 |
| **Mauritania** | 7.96 | 7.41 | 12.06 | 6.41 | 4.04 | 3.51 | 6.16 | 4.48 | 50.40 | 31.67 | 26.82 | 14.48 | 12.96 | 6.14 | 6.67 |
| **Mexico** | 0.04 | 0.25 | 0.29 | 0.30 | 0.38 | 1.17 | 1.08 | 0.31 | 0.33 | 0.11 | 0.31 | 0.04 | 0.05 | 0.37 | 2.59 |
| **Morocco** | 3.87 | 3.21 | 1.82 | 2.72 | 4.20 | 5.52 | 7.08 | 3.98 | 5.57 | 4.27 | 5.12 | 5.82 | 7.91 | 4.60 | 8.73 |
| **Mozambique** | 2.54 | 2.50 | 3.54 | 2.64 | 2.55 | 4.87 | 4.67 | 4.93 | 4.42 | 4.91 | 5.05 | 5.20 | 6.78 | 7.48 | 3.72 |
| **Myanmar** | 0.05 | 0.06 | 0.28 | 0.07 | 0.03 | 0.02 | 0.03 | 0.05 | 0.10 | 0.06 | 0.30 | 0.08 | 0.16 | 0.23 | 0.30 |
| **Nepal** | 1.06 | 1.97 | 1.20 | 1.90 | 0.82 | 0.67 | 1.42 | 1.88 | 1.66 | 1.00 | 1.82 | 1.58 | 1.74 | 2.64 | 2.83 |
| **Niger** | 1.96 | 2.50 | 2.51 | 3.34 | 4.36 | 4.44 | 4.51 | 2.46 | 3.28 | 3.15 | 2.91 | 2.07 | 1.68 | 2.49 | 2.93 |
| **Nigeria** | 0.16 | 0.08 | 0.11 | 0.10 | 0.22 | 0.32 | 0.42 | 0.83 | 0.83 | 0.77 | 0.82 | 0.82 | 0.74 | 0.93 | 1.07 |
| **Pakistan** | 0.45 | 0.51 | 0.24 | 0.18 | 0.30 | 0.45 | 0.73 | 0.80 | 0.41 | 0.40 | 0.42 | 0.45 | 0.45 | 0.28 | 0.30 |
| **Papua New Guinea** | 2.91 | 0.91 | 3.25 | 0.66 | 1.47 | 0.43 | 0.73 | 0.62 | 0.80 | 0.38 | 2.43 | 1.19 | 1.74 | 0.36 | 0.76 |
| **Peru** | 3.90 | 2.24 | 0.84 | 1.06 | 1.52 | 1.66 | 2.42 | 2.89 | 9.78 | 4.53 | 8.00 | 7.87 | 2.14 | 1.90 | 1.75 |
| **Philippines** | 0.72 | 1.03 | 0.21 | 0.35 | 0.35 | 0.26 | 0.28 | 0.21 | 0.43 | 0.27 | 0.30 | 0.12 | 0.07 | 0.13 | 0.17 |
| **Rwanda** | 1.31 | 0.81 | 1.03 | 0.89 | 5.78 | 3.41 | 3.97 | 5.04 | 4.52 | 2.29 | 4.01 | 3.26 | 3.17 | 3.80 | 1.96 |
| **Sao Tome and Principe** | 69.79 | 35.29 | 21.04 | 60.20 | 23.16 | 14.50 | 7.12 | 8.13 | 13.26 | 16.85 | 30.78 | 7.69 | 3.10 | 5.40 | 5.46 |
| **Senegal** | 4.75 | 3.51 | 4.87 | 5.32 | 9.12 | 6.67 | 15.28 | 8.48 | 7.47 | 5.74 | 5.85 | 9.82 | 6.08 | 5.06 | 4.63 |
| **Sierra Leone** | 3.78 | 5.70 | 1.71 | 0.71 | 1.69 | 2.13 | 1.30 | 3.13 | 3.27 | 2.42 | 2.64 | 3.66 | 5.18 | 7.89 | 7.34 |
| **Solomon Islands** | 16.71 | 0.83 | 0.47 | 0.33 | 0.30 | 2.08 | 1.94 | 0.32 | 0.77 | 1.10 | 14.85 | 10.98 | 14.81 | 37.51 | 10.20 |
| **Somalia** | 0.38 | 2.35 | 1.08 | 0.34 | 0.26 | 0.69 | 0.72 | 0.60 | 0.47 | 0.67 | 0.38 | 0.94 | 1.36 | 0.94 | 0.81 |
| **South Africa** | 1.18 | 0.41 | 0.35 | 0.32 | 0.94 | 1.02 | 2.10 | 0.44 | 1.16 | 1.37 | 1.14 | 3.02 | 0.12 | 0.12 | 0.13 |
| **Sudan** | 0.45 | 0.66 | 0.66 | 0.38 | 0.45 | 1.03 | 1.46 | 1.72 | 1.56 | 1.29 | 2.06 | 1.15 | 0.97 | 1.19 | 0.76 |
| **Swaziland** | 2.53 | 1.43 | 0.29 | 1.85 | 3.77 | 2.90 | 0.74 | 0.09 | 1.06 | 0.27 | 0.69 | 0.86 | 0.52 | 1.77 | 2.47 |
| **Tajikistan** | 0.12 | 0.21 | 0.51 | 0.59 | 2.26 | 3.12 | 2.04 | 1.93 | 2.09 | 1.84 | 1.76 | 1.77 | 2.69 | 3.10 | 3.88 |
| **Tanzania** | 2.00 | 1.75 | 1.17 | 2.58 | 1.27 | 1.81 | 4.48 | 5.05 | 4.18 | 5.06 | 5.40 | 3.94 | 5.12 | 3.86 | 3.47 |
| **Togo** | 2.58 | 0.72 | 0.65 | 1.58 | 1.74 | 0.77 | 0.89 | 0.36 | 0.61 | 1.10 | 1.41 | 1.79 | 1.48 | 1.57 | 1.91 |
| **Turkmenistan** | 0.06 | 0.04 | 0.15 | 0.03 | 0.04 | 0.05 | 0.09 | 0.07 | 0.06 | 0.14 | 0.04 | 0.09 | 0.03 | 0.02 | 0.01 |
| **Uganda** | 6.26 | 7.00 | 1.93 | 3.12 | 2.93 | 4.24 | 4.08 | 3.93 | 2.44 | 3.97 | 2.21 | 1.95 | 2.81 | 2.66 | 2.74 |
| **Uzbekistan** | 0.51 | 0.50 | 0.65 | 0.62 | 0.62 | 0.40 | 0.37 | 0.43 | 0.45 | 0.21 | 0.92 | 0.99 | 1.32 | 2.02 | 3.32 |
| **Viet Nam** | 1.43 | 2.18 | 1.26 | 1.59 | 1.55 | 1.83 | 1.33 | 2.71 | 3.37 | 3.99 | 3.59 | 3.69 | 4.47 | 5.52 | 5.33 |
| **Yemen** | 4.71 | 2.85 | 2.37 | 1.69 | 1.72 | 3.91 | 3.33 | 3.54 | 3.30 | 2.65 | 2.41 | 1.28 | 1.28 | 2.53 | 2.12 |
| **Zambia** | 18.32 | 3.97 | 4.30 | 4.59 | 6.08 | 5.84 | 7.83 | 5.26 | 3.48 | 4.48 | 3.16 | 3.90 | 5.47 | 7.24 | 7.57 |
| **Zimbabwe** | 2.28 | 0.96 | 0.21 | 0.18 | 0.07 | 0.11 | 0.16 | 0.02 | 0.96 | 0.70 | 1.38 | 2.74 | 4.86 | 4.12 | 3.80 |

*4. Education*

|  | **2000** | **2001** | **2002** | **2003** | **2004** | **2005** | **2006** | **2007** | **2008** | **2009** | **2010** | **2011** | **2012** | **2013** | **2014** |
| --- | --- | --- | --- | --- | --- | --- | --- | --- | --- | --- | --- | --- | --- | --- | --- |
| **Afghanistan** | 0.32 | 0.56 | 0.83 | 1.15 | 4.00 | 8.17 | 3.50 | 5.69 | 5.06 | 6.33 | 11.37 | 8.81 | 8.17 | 6.70 | 10.89 |
| **Angola** | 0.48 | 0.44 | 0.85 | 1.63 | 0.69 | 1.05 | 1.50 | 1.21 | 1.13 | 1.24 | 0.87 | 0.88 | 1.04 | 0.57 | 0.47 |
| **Azerbaijan** | 0.28 | 0.17 | 0.21 | 0.20 | 0.40 | 0.42 | 1.38 | 1.55 | 0.41 | 0.29 | 0.68 | 0.47 | 0.38 | 0.51 | 0.39 |
| **Bangladesh** | 0.89 | 0.95 | 1.29 | 1.03 | 1.92 | 2.06 | 2.87 | 3.17 | 2.53 | 2.19 | 2.31 | 2.32 | 2.99 | 2.70 | 2.91 |
| **Benin** | 2.65 | 2.53 | 1.65 | 2.90 | 3.70 | 2.16 | 1.88 | 2.41 | 4.00 | 2.61 | 3.04 | 4.23 | 2.64 | 3.03 | 1.69 |
| **Bolivia** | 4.96 | 8.25 | 10.84 | 5.86 | 4.53 | 2.24 | 14.65 | 5.87 | 5.87 | 5.01 | 4.61 | 2.70 | 2.55 | 1.87 | 1.79 |
| **Botswana** | 2.16 | 2.36 | 1.12 | 1.10 | 2.05 | 1.55 | 1.49 | 1.54 | 1.38 | 2.31 | 0.40 | 0.38 | 0.36 | 0.09 | 0.14 |
| **Brazil** | 0.02 | 0.04 | 0.05 | 0.04 | 0.04 | 0.04 | 0.07 | 0.08 | 0.07 | 0.06 | 0.08 | 0.04 | 0.06 | 0.06 | 0.05 |
| **Burkina Faso** | 2.26 | 2.53 | 3.39 | 4.32 | 5.66 | 2.89 | 9.14 | 7.46 | 7.12 | 4.26 | 4.49 | 3.91 | 3.59 | 3.80 | 3.16 |
| **Burundi** | 0.49 | 0.75 | 0.61 | 0.75 | 0.65 | 1.41 | 1.19 | 2.10 | 1.94 | 1.95 | 2.21 | 2.18 | 1.46 | 1.13 | 1.05 |
| **Cambodia** | 2.16 | 1.96 | 0.79 | 1.14 | 2.09 | 2.27 | 3.80 | 3.28 | 2.93 | 2.16 | 2.18 | 2.94 | 2.15 | 3.52 | 2.73 |
| **Cameroon** | 0.98 | 0.45 | 0.87 | 1.06 | 1.25 | 1.10 | 1.19 | 2.29 | 0.84 | 0.48 | 0.66 | 1.10 | 1.30 | 0.61 | 0.74 |
| **Central African Republic** | 1.00 | 0.43 | 0.46 | 0.44 | 0.60 | 0.76 | 0.40 | 0.54 | 0.40 | 0.92 | 0.99 | 0.80 | 0.35 | 0.14 | 0.01 |
| **Chad** | 2.00 | 1.82 | 1.21 | 0.72 | 0.75 | 1.39 | 0.91 | 1.08 | 0.96 | 0.95 | 1.30 | 0.95 | 0.96 | 0.56 | 0.56 |
| **China** | 0.06 | 0.04 | 0.03 | 0.04 | 0.05 | 0.05 | 0.06 | 0.03 | 0.02 | 0.02 | 0.02 | 0.02 | 0.01 | 0.02 | 0.04 |
| **Comoros** | 2.96 | 8.31 | 10.20 | 6.65 | 1.53 | 0.29 | 1.08 | 0.93 | 0.79 | 0.93 | 1.04 | 1.94 | 6.51 | 1.79 | 2.57 |
| **Congo** | 0.17 | 0.23 | 0.26 | 0.34 | 0.59 | 1.60 | 2.20 | 2.83 | 3.53 | 1.26 | 2.06 | 1.84 | 2.03 | 1.31 | 1.23 |
| **Cote d'Ivoire** | 0.79 | 0.67 | 0.83 | 0.66 | 0.49 | 0.42 | 0.42 | 0.66 | 0.47 | 0.66 | 1.95 | 0.52 | 1.23 | 0.76 | 0.79 |
| **DPR Korea** | 0.02 | 0.03 | 0.04 | 0.07 | 0.06 | 0.06 | 0.03 | 0.03 | 0.04 | 0.06 | 0.10 | 0.03 | 0.08 | 0.08 | 0.01 |
| **DR Congo** | 0.16 | 0.26 | 0.26 | 0.25 | 0.23 | 0.21 | 0.38 | 0.70 | 0.93 | 0.96 | 1.39 | 1.10 | 1.30 | 1.55 | 1.10 |
| **Djibouti** | 30.14 | 21.58 | 17.74 | 17.90 | 17.85 | 21.85 | 13.53 | 12.98 | 12.70 | 11.38 | 10.20 | 9.52 | 12.44 | 11.68 | 5.25 |
| **Egypt** | 0.96 | 1.18 | 0.95 | 1.18 | 1.61 | 0.75 | 1.81 | 2.13 | 2.24 | 1.31 | 0.97 | 0.59 | 0.88 | 0.58 | 0.46 |
| **Equatorial Guinea** | 10.44 | 1.82 | 11.40 | 4.15 | 18.61 | 6.07 | 1.53 | 0.53 | 0.95 | 1.10 | 4.58 | 6.42 | 4.09 | 0.53 | 0.36 |
| **Eritrea** | 4.19 | 3.03 | 1.86 | 2.49 | 3.26 | 3.42 | 1.74 | 8.10 | 2.45 | 1.23 | 2.04 | 4.05 | 0.49 | 2.44 | 0.77 |
| **Ethiopia** | 1.84 | 0.93 | 0.50 | 0.84 | 1.14 | 0.61 | 2.85 | 3.39 | 0.92 | 0.83 | 0.84 | 0.85 | 0.89 | 1.62 | 2.02 |
| **Gabon** | 6.09 | 7.16 | 8.25 | 12.33 | 8.64 | 1.80 | 1.51 | 1.42 | 2.14 | 2.89 | 3.98 | 2.58 | 1.76 | 1.63 | 1.91 |
| **Gambia** | 5.57 | 4.70 | 5.37 | 3.49 | 3.22 | 4.79 | 2.63 | 23.82 | 6.34 | 2.12 | 5.90 | 4.39 | 2.98 | 2.10 | 1.37 |
| **Ghana** | 4.98 | 3.73 | 2.94 | 3.12 | 3.10 | 4.16 | 3.22 | 2.96 | 2.38 | 1.89 | 2.17 | 1.99 | 2.00 | 4.55 | 3.10 |
| **Guatemala** | 3.42 | 2.29 | 2.15 | 1.50 | 1.73 | 1.63 | 1.85 | 1.15 | 2.34 | 1.61 | 2.22 | 1.71 | 1.93 | 1.58 | 1.52 |
| **Guinea** | 2.39 | 2.30 | 3.25 | 3.86 | 4.37 | 4.74 | 4.19 | 3.40 | 2.47 | 1.07 | 1.13 | 0.86 | 2.06 | 0.80 | 1.06 |
| **Guinea-Bissau** | 3.02 | 3.28 | 4.35 | 4.02 | 5.89 | 3.31 | 2.44 | 3.36 | 8.12 | 3.99 | 3.72 | 8.87 | 2.58 | 4.00 | 3.36 |
| **Haiti** | 1.84 | 1.99 | 1.99 | 1.87 | 2.67 | 1.70 | 1.84 | 1.75 | 3.18 | 6.34 | 10.36 | 9.62 | 5.00 | 4.76 | 5.48 |
| **India** | 0.27 | 0.36 | 0.24 | 0.32 | 0.38 | 0.69 | 0.53 | 0.12 | 0.31 | 0.58 | 0.41 | 0.53 | 0.13 | 0.26 | 0.67 |
| **Indonesia** | 0.51 | 0.80 | 0.37 | 0.20 | 0.21 | 0.24 | 0.56 | 0.49 | 1.02 | 1.14 | 0.77 | 0.44 | 0.22 | 0.25 | 0.17 |
| **Iraq** | 0.05 | 0.03 | 0.03 | 0.48 | 2.41 | 5.56 | 0.23 | 0.47 | 0.21 | 0.74 | 1.00 | 0.56 | 1.76 | 1.22 | 1.07 |
| **Kenya** | 0.95 | 0.73 | 0.80 | 3.49 | 1.76 | 1.51 | 0.71 | 2.12 | 1.89 | 1.20 | 0.75 | 1.23 | 1.39 | 1.69 | 1.79 |
| **Kyrgyzstan** | 0.26 | 0.45 | 0.34 | 0.90 | 0.77 | 0.94 | 3.60 | 2.89 | 2.87 | 2.87 | 3.72 | 6.88 | 3.96 | 1.87 | 2.97 |
| **Lao** | 1.17 | 1.79 | 3.49 | 2.21 | 3.13 | 3.08 | 6.32 | 3.94 | 7.93 | 5.74 | 6.09 | 5.93 | 6.56 | 8.33 | 13.16 |
| **Lesotho** | 2.24 | 3.38 | 9.31 | 6.67 | 5.61 | 9.62 | 6.64 | 5.24 | 8.51 | 6.40 | 2.88 | 7.80 | 1.33 | 1.30 | 1.23 |
| **Liberia** | 1.36 | 0.70 | 0.98 | 1.47 | 1.65 | 1.19 | 0.78 | 2.12 | 4.55 | 7.28 | 9.73 | 6.50 | 5.35 | 5.66 | 4.55 |
| **Madagascar** | 0.70 | 0.92 | 1.17 | 1.94 | 1.09 | 1.45 | 4.13 | 2.82 | 1.59 | 1.61 | 1.48 | 1.24 | 1.14 | 1.85 | 1.06 |
| **Malawi** | 6.52 | 5.31 | 5.35 | 3.62 | 3.74 | 3.07 | 8.34 | 3.44 | 2.98 | 4.13 | 6.65 | 3.98 | 5.01 | 3.09 | 4.31 |
| **Mali** | 6.08 | 3.18 | 2.50 | 4.36 | 7.20 | 4.31 | 6.68 | 6.53 | 8.62 | 7.02 | 7.38 | 4.77 | 3.02 | 2.09 | 2.56 |
| **Mauritania** | 10.32 | 3.95 | 4.86 | 3.90 | 1.77 | 3.83 | 4.99 | 9.29 | 4.85 | 2.96 | 3.59 | 3.92 | 2.43 | 1.62 | 1.74 |
| **Mexico** | 0.02 | 0.03 | 0.10 | 0.06 | 0.04 | 0.04 | 0.09 | 0.10 | 0.08 | 0.07 | 0.11 | 0.07 | 0.07 | 0.07 | 0.08 |
| **Morocco** | 0.61 | 0.38 | 0.32 | 0.54 | 1.35 | 1.45 | 3.46 | 3.09 | 3.60 | 3.07 | 2.92 | 3.51 | 3.21 | 3.90 | 2.58 |
| **Mozambique** | 2.46 | 3.35 | 4.03 | 3.48 | 3.31 | 4.42 | 6.43 | 4.73 | 4.98 | 5.54 | 3.58 | 3.34 | 2.84 | 3.19 | 3.45 |
| **Myanmar** | 0.04 | 0.04 | 0.07 | 0.17 | 0.08 | 0.12 | 0.13 | 0.34 | 0.46 | 0.35 | 0.49 | 0.58 | 1.07 | 0.77 | 1.49 |
| **Nepal** | 2.17 | 1.94 | 0.98 | 3.04 | 2.51 | 2.18 | 3.72 | 4.08 | 3.61 | 5.49 | 2.83 | 1.94 | 2.81 | 3.10 | 4.12 |
| **Niger** | 1.40 | 1.40 | 1.26 | 1.37 | 1.95 | 2.55 | 2.61 | 3.93 | 2.47 | 1.66 | 1.13 | 0.76 | 1.22 | 1.46 | 1.78 |
| **Nigeria** | 0.45 | 0.08 | 0.08 | 0.18 | 0.29 | 0.42 | 0.43 | 0.56 | 0.23 | 0.30 | 0.49 | 0.37 | 0.43 | 0.58 | 0.38 |
| **Pakistan** | 0.92 | 1.58 | 0.39 | 0.33 | 0.24 | 0.45 | 0.68 | 2.51 | 0.91 | 1.33 | 1.05 | 0.79 | 0.97 | 1.13 | 1.47 |
| **Papua New Guinea** | 11.63 | 12.19 | 7.63 | 4.68 | 3.85 | 4.69 | 4.81 | 2.26 | 3.08 | 7.02 | 8.64 | 8.48 | 0.68 | 0.58 | 0.79 |
| **Peru** | 0.95 | 0.61 | 0.70 | 0.78 | 0.92 | 1.03 | 0.94 | 1.34 | 1.10 | 1.41 | 0.97 | 0.73 | 0.67 | 0.56 | 0.72 |
| **Philippines** | 0.36 | 0.25 | 0.15 | 0.18 | 0.33 | 0.61 | 0.37 | 0.18 | 0.61 | 0.28 | 0.45 | 0.27 | 0.48 | 0.37 | 0.59 |
| **Rwanda** | 1.70 | 2.95 | 1.27 | 1.80 | 1.83 | 2.39 | 2.76 | 2.59 | 5.47 | 6.41 | 2.51 | 3.84 | 2.27 | 3.30 | 3.75 |
| **Sao Tome and Principe** | 13.23 | 31.64 | 28.20 | 16.05 | 13.16 | 15.10 | 19.84 | 17.81 | 16.85 | 9.67 | 17.24 | 20.02 | 28.05 | 15.55 | 12.59 |
| **Senegal** | 3.64 | 1.84 | 2.00 | 4.00 | 5.77 | 4.09 | 4.18 | 5.12 | 5.38 | 4.29 | 5.10 | 5.16 | 6.21 | 4.88 | 5.99 |
| **Sierra Leone** | 0.32 | 1.52 | 3.11 | 2.29 | 3.21 | 4.22 | 2.73 | 3.80 | 4.69 | 3.76 | 2.27 | 4.29 | 3.07 | 2.69 | 3.10 |
| **Solomon Islands** | 16.68 | 22.51 | 2.76 | 4.39 | 30.70 | 23.64 | 31.62 | 17.99 | 27.17 | 38.46 | 27.12 | 35.66 | 37.51 | 45.31 | 22.86 |
| **Somalia** | 0.19 | 0.17 | 0.40 | 0.48 | 0.41 | 0.70 | 0.94 | 0.72 | 2.26 | 3.38 | 4.06 | 3.55 | 4.38 | 2.89 | 2.19 |
| **South Africa** | 1.35 | 1.24 | 1.34 | 1.74 | 1.28 | 1.34 | 1.50 | 1.70 | 0.85 | 0.93 | 1.96 | 1.32 | 1.37 | 0.26 | 0.28 |
| **Sudan** | 0.10 | 0.28 | 0.24 | 0.38 | 0.41 | 0.44 | 0.84 | 0.93 | 1.57 | 1.98 | 1.42 | 1.42 | 0.55 | 0.37 | 0.40 |
| **Swaziland** | 0.65 | 0.69 | 0.35 | 0.97 | 1.27 | 5.05 | 1.96 | 4.13 | 3.07 | 2.12 | 7.24 | 14.97 | 5.96 | 3.88 | 4.43 |
| **Tajikistan** | 0.94 | 1.01 | 0.70 | 0.32 | 0.65 | 1.11 | 1.37 | 1.57 | 1.69 | 2.62 | 2.58 | 2.71 | 2.48 | 1.09 | 1.01 |
| **Tanzania** | 3.80 | 2.15 | 5.64 | 6.55 | 8.14 | 1.92 | 8.25 | 3.29 | 1.62 | 1.43 | 2.22 | 0.91 | 2.05 | 1.54 | 0.90 |
| **Togo** | 0.65 | 0.79 | 0.55 | 0.14 | 0.49 | 0.57 | 1.40 | 1.01 | 0.81 | 1.30 | 1.87 | 2.64 | 0.83 | 1.27 | 1.87 |
| **Turkmenistan** | 0.24 | 0.16 | 0.17 | 0.11 | 0.26 | 0.25 | 0.56 | 0.39 | 0.19 | 0.25 | 0.53 | 0.42 | 0.44 | 0.40 | 0.34 |
| **Uganda** | 5.12 | 3.25 | 6.87 | 3.55 | 3.09 | 2.34 | 2.70 | 2.10 | 3.31 | 1.62 | 4.43 | 1.55 | 3.11 | 3.45 | 2.99 |
| **Uzbekistan** | 0.56 | 0.54 | 0.24 | 0.22 | 2.20 | 0.84 | 0.35 | 0.39 | 0.51 | 0.59 | 1.85 | 1.33 | 1.18 | 0.47 | 0.27 |
| **Viet Nam** | 0.43 | 0.49 | 0.44 | 0.43 | 0.77 | 0.44 | 0.74 | 2.07 | 1.64 | 1.86 | 2.15 | 1.20 | 1.19 | 1.24 | 1.51 |
| **Yemen** | 1.51 | 1.67 | 1.03 | 1.62 | 2.85 | 2.36 | 2.62 | 3.51 | 2.64 | 3.27 | 3.16 | 2.37 | 2.21 | 1.83 | 1.47 |
| **Zambia** | 4.82 | 4.59 | 6.62 | 4.38 | 5.63 | 3.99 | 7.49 | 5.59 | 3.18 | 1.99 | 1.49 | 1.22 | 1.55 | 1.58 | 1.65 |
| **Zimbabwe** | 0.25 | 0.53 | 0.52 | 0.28 | 0.23 | 0.20 | 0.18 | 0.16 | 0.18 | 0.45 | 0.61 | 1.12 | 4.45 | 3.30 | 3.56 |
