# Supplementary material for: Assessing development assistance for child survival between 2000 and 2014: A multi-sectoral perspective
Source: PLoS One. 2017 Jul 11;12(7):e0178887. doi: 10.1371/journal.pone.0178887 (PMC5507412; doi:10.1371/journal.pone.0178887)
Supplement: S2 Fig — (DOCX) [file pone.0178887.s013.docx]

**S2 Fig.** Missing rate for disbursements by income group in CRS
