# Supplementary material for: Assessing development assistance for child survival between 2000 and 2014: A multi-sectoral perspective
Source: PLoS One. 2017 Jul 11;12(7):e0178887. doi: 10.1371/journal.pone.0178887 (PMC5507412; doi:10.1371/journal.pone.0178887)
Supplement: S9 Fig — (DOCX) [file pone.0178887.s020.docx]

**S9 Fig.** Global trends of aid disbursed to health, RMNCH, food and humanitarian assistance, water and sanitation, and education in 134 countries with six sets of estimates, 2000-2014 (in millions of 2013 USD)

CRS: estimates from CRS data

EST: estimates from CRS + DIF (difference between CRS and DAC2)

CRS__rgn_: estimates from CRS data + allocated regional funds

EST__rgn_: estimates from CRS + DIF (difference between CRS and DAC2) + allocated regional funds

CRS__rgn_unsp_: estimates from CRS data + allocated regional funds + allocated funds labelled as “developing countries”

EST__rgn_unsp_: estimates from CRS + DIF (difference between CRS and DAC2) + allocated regional funds + allocated funds labelled as “developing countries”

1. Aid disbursed to health
2. Aid disbursed to food and humanitarian assistance
3. Aid disbursed to water and sanitation
4. Aid disbursed to education
5. Aid disbursed to RMNCH: upper bound
6. Aid disbursed to RMNCH: lower bound (excluding HIV/TB/health system strengthening)
