# Supplementary material for: Assessing development assistance for child survival between 2000 and 2014: A multi-sectoral perspective
Source: PLoS One. 2017 Jul 11;12(7):e0178887. doi: 10.1371/journal.pone.0178887 (PMC5507412; doi:10.1371/journal.pone.0178887)
Supplement: S13 Fig — (DOCX) [file pone.0178887.s024.docx]

**S13 Fig.** Upper-bound estimates of aid disbursed to RMNCH, health_non-RMNCH, food, water and sanitation, humanitarian assistance, and education in billions in 2013 USD, Countdown vs. non-Countdown, 2000-2014
